# Coarse-grained modelling to predict the packing of porous organic cages†

**DOI:** 10.1039/d2sc04511g

**Published:** 2022-10-25

**Authors:** Emma H. Wolpert, Kim E. Jelfs

**Affiliations:** Department of Chemistry, Imperial College London, Molecular Sciences Research Hub White City Campus, Wood Lane London W12 0BZ UK e.wolpert@imperial.ac.uk k.jelfs@imperial.ac.uk +44 (0)20759 43438

## Abstract

How molecules pack has vital ramifications for their applications as functional molecular materials. Small changes in a molecule's functionality can lead to large, non-intuitive, changes in their global solid-state packing, resulting in difficulty in targeted design. Predicting the crystal structure of organic molecules from only their molecular structure is a well-known problem plaguing crystal engineering. Although relevant to the properties of many organic molecules, the packing behaviour of modular porous materials, such as porous organic cages (POCs), greatly impacts the properties of the material. We present a novel way of predicting the solid-state phase behaviour of POCs by using a simplistic model containing the dominant degrees of freedom driving crystalline phase formation. We employ coarse-grained simulations to systematically study how chemical functionality of pseudo-octahedral cages can be used to manipulate the solid-state phase formation of POCs. Our results support those of experimentally reported structures, showing that for cages which pack *via* their windows forming a porous network, only one phase is formed, whereas when cages pack *via* their windows and arenes, the phase behaviour is more complex. While presenting a lower computational cost route for predicting molecular crystal packing, coarse-grained models also allow for the development of design rules which we start to formulate through our results.

## Abstract 2

This work presents a novel method for predicting molecular crystal structure formation using coarse-grained modelling, enabling the development of design rules.

## Graphical abstract

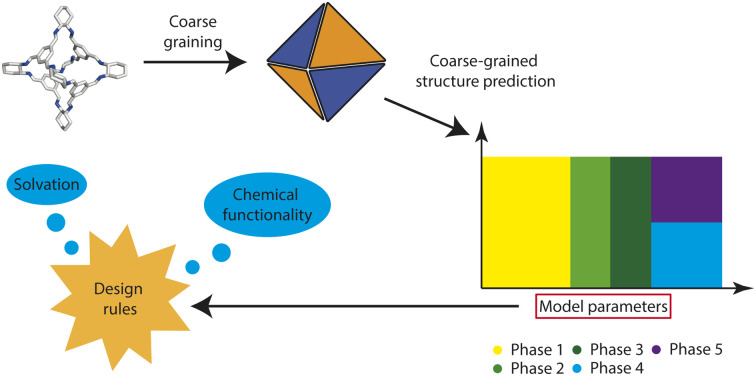

## 1 Introduction

How molecules pack has vital ramifications for their application in areas such as optoelectronics,^1^ catalysis,^2^ and drug delivery.^3^ However, the lack of strong bonds has resulted in the long-standing challenge of how to design molecules to manipulate crystal formation for specific packings with desired properties.^4^ The prediction of solid-state packing is non-trivial and is more complex for molecular materials than their inorganic counterparts. Reticular design rules implemented in extended framework formation such as metal- and covalent-organic frameworks fail when applied to organic molecules due to their packing being determined by weak dispersion forces.

Currently, state-of-the-art computational efforts focus on crystal structure prediction (CSP) which, although successful,^5–7^ is computationally expensive and must be employed on a case-by-case basis. This results in difficulty formulating design rules between structural motifs, chemical functionality, and their packing behaviour. Moreover, weak intermolecular interactions result in a high number of possible low-energy polymorphs and, coupled with the high computational cost of these calculations,^8^ this restricts the ability to use high-throughput approaches.^5^ Additionally, it is even more computationally expensive to apply CSP to multicomponent systems,^9^ where it has been shown that combining different molecules can allow property tuning in a way that is not possible with extended framework materials.^10^ Therefore, a novel method of predicting the packing of molecular materials must be introduced in order to simultaneously improve our understanding of molecular crystals, reduce computational efforts, and elucidate design rules for targeted phase formation.

**Fig. 1:**
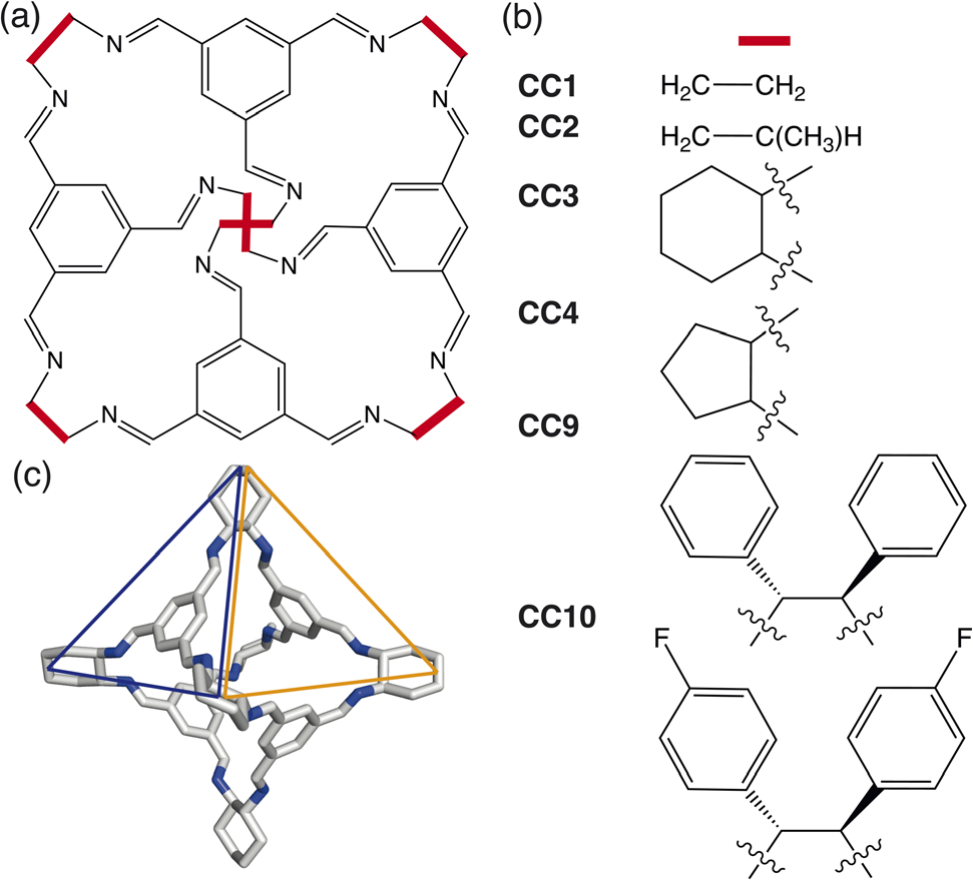
(a) Basic structure of a POC formed from the condensation of a trialdehyde and diamine in a [4 + 6] cycloimination where the vertices have different functional groups depending on the diamine used. (b) Examples of the different chemical functionalities of common octahedral POCs reported within the literature. (c) An example of the 3D structure of the octahedral POC CC3 which has pseudo-tetrahedral symmetry due to the different chemical features of the octahedral cage where there is either an arene (blue) or a window (orange) at the centre of the octahedral face. The carbon/nitrogen atoms are shown in white/blue and the hydrogens atoms are omitted for clarity.

In this paper, we develop a novel way of predicting the molecular solid-state based on a simplistic, coarse-grained model containing the dominant degrees of freedom driving crystalline phase formation. We focus on determining the packing behaviour of a subset of porous organic cages (POCs) formed through a condensation reaction between a trialdehyde and diamine in a [4 + 6] cycloimination, creating discrete organic molecules which contain a permanent internal cavity (Fig. 1). Although the cages formed are pseudo-octahedral geometrically, the molecules have pseudo-tetrahedral symmetry due to the different chemical features of the cages, where each face of the octahedra is either a cavity, known as a window, or contains an arene (Fig. 1(c)). The porous nature of POCs leads to a variety of potential applications, including in catalysis,^11^ sensing,^12^ encapsulation^13^ and molecules separations.^14^ Despite significant interest in POCs, their utilisation in industrial applications is hindered by the lack of understanding of how to control their assembly,^15^ both on a molecular level and in the solid-state. As techniques for predicting the molecular assembly of cage molecules become more refined,^16,17^ there still remains the question of how to predict and control the assembly in the solid-state.

**Fig. 2:**
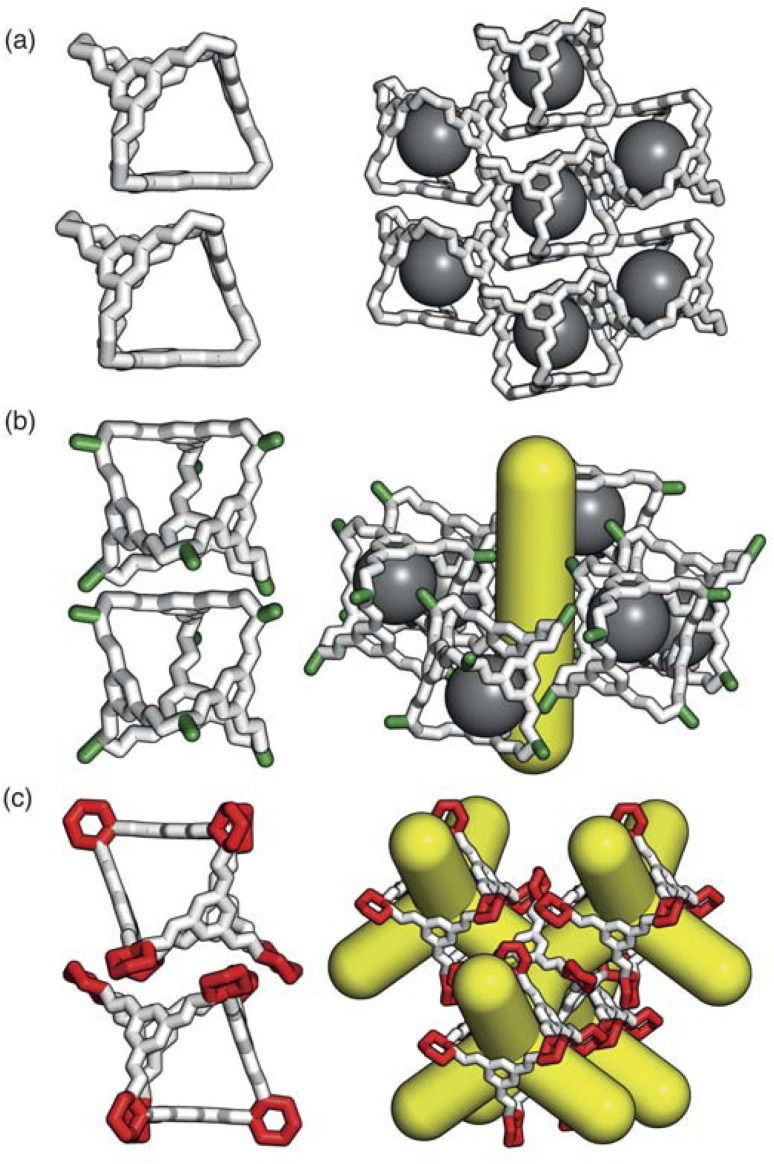
The different functionalities of the vertices of the cages lead to vastly different packing arrangements, affecting the pore networks of the material as highlighted through (a) the 0D pore network formed by CC1α′, (b) the 1D pore network formed by CC2 due to the methyl functionality (green), and (c) the 3D pore network formed by CC3α due to the cyclohexyl groups (red). Isolated voids formed by window-to-arene packings are shown in grey and the pore networks are shown in yellow. Both the carbon and nitrogen atoms are shown in white and the hydrogen atoms are omitted for clarity.

As the internal cavity of the cages can only be accessed through their windows, changes in their solid-state phase behaviour can have a significant affect on the pore network in the material. Small changes in the chemical functionality of POCs can lead to non-intuitive changes in the global solid-state packing due to the fine balance between the weak dispersion forces (Fig. 2). These weak interactions are responsible for many of the advantages of POCs over extended frameworks such as their solution processability^18^ and the potential to control their solid-state assembly by manipulating the functionality of the cages or solvent used.^19,20^ However, facile prediction and control of the packing behaviour of novel cages is currently intractable as there remains a lack of understanding of how chemical functionality and solvent interplay in the formation of molecular solid-state materials. This complexity in predicting the packing behaviour of molecular crystals makes targeted design difficult.^21^

**Fig. 3:**
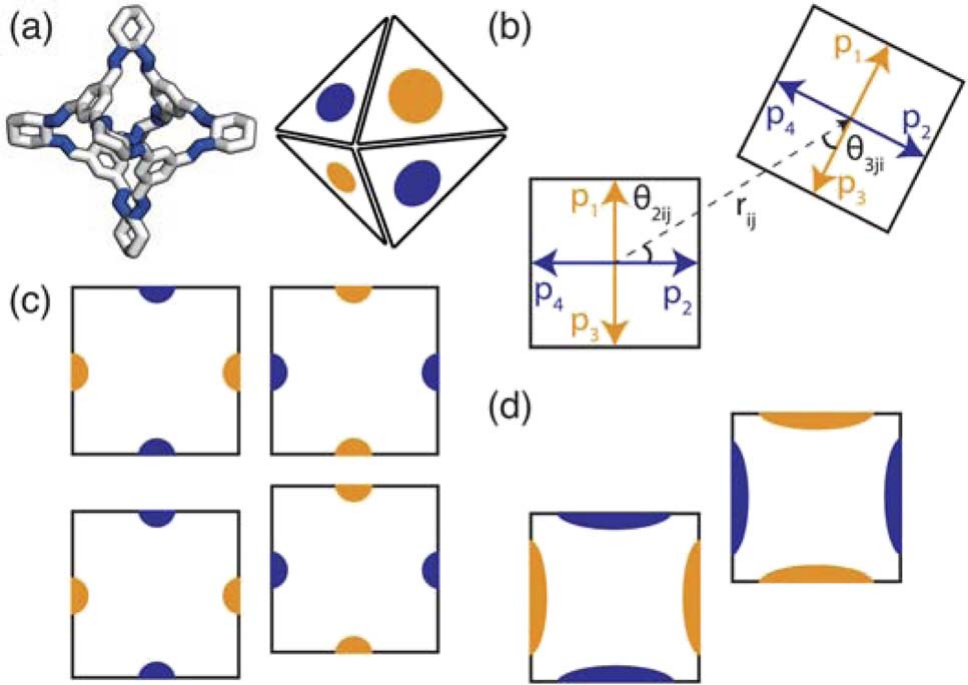
(a) How the POC CC3 relates to the coarse-grained model where the orange and blue patches represent the different chemical nature of the POC's facets (*i.e.* window or arene face). Here the carbon/nitrogen atoms are shown in white/blue and the hydrogens atoms are omitted for clarity. (b–d) Two-dimensional representation of the patchy particle model used. (b) A representation of the geometry of the interaction between two particles for a window-to-arene simulation. Here each of the particles have four patches described by the patch vectors *p*_*α*_ and *p*_*β*_ where *α* and *β* refer to patch *α*(*β*) on atom *i*(*j*). In the particular case shown in the figure, patch 2 on particle *i* interacts with patch 3 on particle *j* as they are the closest patches to the interparticle vector, *r*_*ij*_. To relate the patches to our model we have coloured the patch vectors according to the types of patches. (c) Top: an example of the lowest energy orientation of an interaction with preferred alignment of opposing patches. Bottom: the maximum displacement between the two particles that is likely to occur with a small patch width (small *σ*_ang_). (d) The maximum displacement between the two particles that is likely to occur with a large patch width (large *σ*_ang_) leading to the formation of more ordered structures when *σ*_ang_ is small than for larger values of *σ*_ang_.

To implement the research presented in this paper, we use a general-purpose particle simulation toolkit, HOOMD-blue,^22^ to employ hard particle Monte Carlo (HPMC) simulations with directional interactions through “patches” (Fig. 3(a)). HPMC simulations as implemented within HOOMD-blue are often used to investigate the self assembly of structures formed by packing non-interacting polyhedra.^23,24^ Through these simulations, Glotzer and coworkers have shown that geometry alone can direct complex structural formation through an “entropic bond” governed by the polyhedral shape. The entropic bond is a purely statistical phenomena which explains that densely packed structures form from non-interacting particles due to the system maximising the number of microstates available. While the studies focused on colloidal, non-interacting particles, this has strong implications for packing in molecular materials. The entropic bonds are weak,^25^ on the order of a few *k*_B_*T*,^26^ which means enthalpic interactions dominate in most materials. However, the packing of organic molecules is dictated by weak intermolecular interactions, suggesting that the entropy gained from increasing the number of microstates available may influence the phase behaviour.

Within HOOMD-blue, patches can be added to hard particles in order to mimic directional interactions,^27^ which has been successfully applied to molecular materials as seen in the investigation of the self assembly of π-conjugated optoelectronic peptides.^28^ The benefit of using HOOMD-blue is the ease in using user-defined potentials as required for the introduction of coarse-grained models. This was demonstrated by Mansbach *et al.* who tracked the effects of core and side chain interaction strength and sterics on the morphology and kinetics of assembly using a coarse-grained model.^28^ These models allow for the formation of design rules by developing an understanding of the interactions and geometric building blocks required for targeted phase behaviour.^29^

Here we use HPMC simulations in conjunction with patchy particle models to investigate the effect of the combination of building block geometry and directional interactions on the solid-state phase behaviour of POCs. The basic framework rigidity of POCs means that geometrically, the molecules can be considered as polyhedra. For the POCs considered in this paper (Fig. 1), that means we can represent the molecular cages as hard octahedra for the HPMC simulations. The directional interactions are introduced through patches on each of the facets, where the different colour patches represent the reduction of symmetry of the octahedra due to the chemical makeup of the cages (*i.e.* the alternation of facets between windows or arenes) (Fig. 3(a)). Due to the simplicity of the model, this procedure drastically reduces computational efforts compared to CSP and facilitates the development of design rules required for targeted phase formation in the future. Although a simplistic model, we show that by manipulating the parameters of our coarse-grained model, we can reproduce the phase space spanned by the majority of octahedral POCs present in the Cambridge Structural Database.^30^ Finally, our results highlight new phases which are unreported within the literature. While the focus of this paper is on POCs, many of the motifs studied here are seen in other areas of molecular chemistry such as metal–organic cages.^31,32^ Thus our results may have wider reaching applications to predict and rationalise the solid-state behaviour of a range of molecular materials.

## 2 Methods

### 2.1 Hard particle Monte Carlo simulations

The Monte Carlo simulations were performed using HPMC,^33^ a plugin to the HOOMD-blue simulation toolkit.^22^ The Metropolis Monte Carlo algorithm in HPMC works by performing moves of either a translation or rotation of the particles. If the move leads to no overlap between any hard particles, it is accepted, whereas if an overlap between neighbouring particles occurs, then the move is rejected. Therefore the simulations are often deemed to be temperature independent as each move either leads to no change in energy, or an infinite increase.^33^ However, in our case we applied interactions between neighbouring particles which caused the simulation results to be affected by temperature, modifying the acceptance rules based on the energetic penalty of a given move determined by the interaction potential.

### 2.2 Patchy particle potential

To determine the phase behaviour spanned by pseudo-octahedral cages, we implemented HPMC simulations on hard octahedra with different directional interactions introduced through patches on the polyhedra. The form of these interactions is based on the patchy particle potential described in ref. 34, which decorates the particle with a number of sticky patches, reducing its symmetry. As shown in Fig. 3(a), a patch was added to the centre of each facet (*i.e.* window or arene face), where the different colour patches represent the reduction of symmetry of the octahedra due to the chemical makeup of the cages (*i.e.* the alternation of facets between windows or arenes). To include the likely degrees of freedom that occur between POCs, the patches on the hard octahedra were introduced *via* an interaction term, *V*_*ij*_, which contains three components; an attractive Lennard-Jones potential *V*_LJ_, an angular modulation term *V*_ang_, and a torsional modulation term *V*_tor_:1
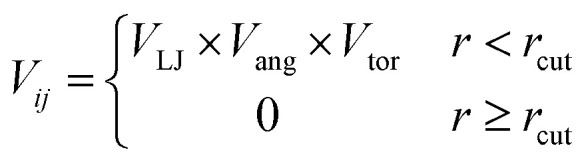
2
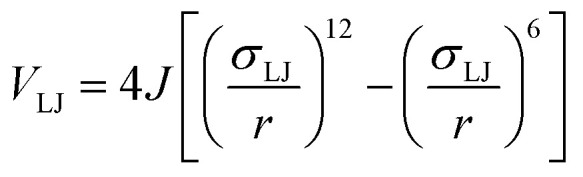
3
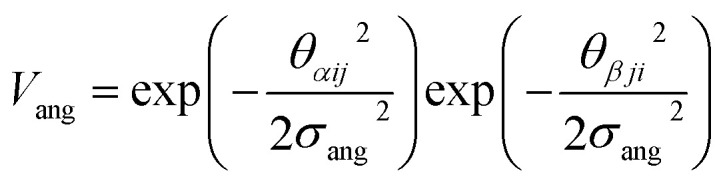
4
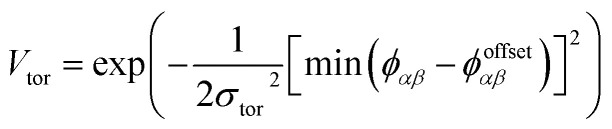
where *α* and *β* are patches on particles *i* and *j* respectively, Fig. 3(b). Throughout the simulations, *σ*_LJ_, which is normally the measure of the diameter of the interacting particles, was set to 0.6 Å. As each octahedra had unit length, this value is slightly larger than the minimum distance required for the two hard octahedra packed along the facets (0.5 Å). This is to allow for a small gap between the hard octahedra, which is likely to exist between the analogous two neighbouring cages due to their van der Waals radii. The cutoff distance, *r*_cut_, is set to 1.8*σ*_LJ_ so that the patches interact with nearest neighbour polyhedra only as *r* is a measure of the distance between the centres of the two neighbouring particles.

The angular term, *V*_ang_, is a measure of how directly the patches on adjacent particles point at each other. Here, *θ*_*αij*_ is the angle between the patch vector and the vector between the two neighbouring particles, **r**_*ij*_, the magnitude of which is given by *r* (Fig. 3(b)). *σ*_ang_ dictates the energetic penalty of particles deviating from being perfectly aligned, such that a larger *σ*_ang_ value relates to worse alignment of the two octahedral facets, Fig. 3(c and d). *σ*_ang_ is referred to as the width of the patch as changing *σ*_ang_ narrows or widens the Gaussian function that describes the patchy interaction, effectively changing the angular width of the patch where the particle interacts through. Through this work, we examine how changing *σ*_ang_ between 0.1 and 1.0 effects the solid-state phase behaviour of the interacting octahedral particles. We expect that this parameter encapsulates the effects of different substituents on the vertices as the substituents would likely change the directionality of the interaction due to steric effects, which is controlled by the patch width.

The torsional term, *V*_tor_, describes the variation in the potential with rotation of the particle about the interparticle vector **r**_*ij*_. *ϕ*^offset^_*αβ*_ is the preferred torsional angle between patches *α* and *β*, whereas *ϕ*_*αβ*_ is the actual torsional angle between patches *α* and *β*, such that there is an energy minimum where the two angles are the same. The preferred torsional angles used for each simulation are summarised in Table S3† and were chosen based on the likely lowest energy orientations of window-to-window and window-to-arene packings due to steric interactions, *i.e.* window-to-window prefers to be anti-aligned and window-to-arene prefers to be aligned. Similarly to *σ*_ang_, *σ*_tor_ dictates the energetic penalty of the particles deviation from the perfect torsional angle. In our simulations, we found that the results did not vary with *σ*_tor_, and as such, much like other studies on patchy particles,^35^ we kept a constant ratio between *σ*_tor_ and *σ*_ang_ such that *σ*_tor_ = 2*σ*_ang_. This is a physically reasonable approximation as, from chemical considerations, it is likely that *σ*_tor_ and *σ*_ang_ are coupled such that the less the interaction cares about the alignment of the two molecules, the less likely it has a strong torsional preference *i.e.* the interaction overall will be less directional.

*J* is the measure of the interaction strength between the two octahedra. Varying *σ*_ang_ in our model causes the clusters to be formed at different temperatures for the same value of *J*. This is because a smaller value of *σ*_ang_ narrows the Gaussian function in eqn (3), resulting in a smaller value of *V*_ang_. As the absolute energy scale is not important to the results of this study, for simplicity a value of *J* was chosen for each simulation such that the transition temperature *T*_t_ occurred when *k*_B_*T*_t_ ≈ 1. The interaction parameters used are summarised in Tables S1 and S2.† We note that for a given value of *σ*_ang_, *J* is larger for the window-to-window simulations than for the window-to-arene simulations. This is because each cage only has four windows, resulting in a maximum of four stabilising window-to-window “interactions” for each cage, whereas in the window-to-arene simulations, there can be up to eight stabilising interactions as the cages can be stabilised through interactions through the arene as well. This means that *J* must be larger to result in a transition occurring at the same temperature.

### 2.3 Simulation details

The simulations were slowly cooled in the NVT ensemble over the transition point, *k*_B_*T* ≈ 1, starting at *k*_B_*T* = 1.35, to ensure full equilibration at steps of *T*_*i*+1_ = *T*_*i*_ × 0.95 where *k*_B_ = 0.00831 kJ mol^−1^ K^−1^. Each simulation was run for 24 hours using 64 cores on ARCHER2 UK National Supercomputing Service, with the exception of the window-to-window simulations where *σ*_ang_ = 0.4 and 0.8, which were run using Imperial College London's Research Computing Service until *k*_B_*T* = 0.51. During the simulations, at least nine temperatures were sampled with 10^8^ timesteps—where one trial move is applied to a random number of particles in each cell during one timestep—taken at every temperature. The time taken to complete a timestep depends on the number of interactions being calculated, therefore once the polyhedra form a cluster, the time taken to complete each timestep increases. Consequently, due to the finite wall time, some simulations, particularly those at low *σ*_ang_, have a fewer number of temperature points simulated. For these cases, once the simulations were terminated, the resulting configuration is considered to be representative of the phase behaviour at all lower temperatures. This assumption is corroborated by results from the window-to-window simulations where *σ*_ang_ = 0.4 and 0.8, as both the ordered state at *σ*_ang_ = 0.4 and amorphous phase at *σ*_ang_ = 0.8 persisted down to *k*_B_*T* = 0.51.

The simulations were performed on 512 particles of unit length in cubic boxes with a box length of 12 Å and periodic boundary conditions. As the potentials are attractive and the systems were initialised at low density, the simulations formed compact clusters with no mechanical stresses and structure defects that are often seen in bulk simulations. To determine if there were any finite size effects, some simulations were repeated with 4096 particles and evidenced the same results.

### 2.4 Phase determination

For a structure to be considered a representative phase at any given simulation condition, we imposed a minimum cluster limit of 50 polyhedra before undergoing any further analysis. To ensure we were sampling from the bulk structure, the outer layer of the cluster is then removed and clusters that still contained over 50 polyhedra underwent subsequent analysis. This limit was chosen as the clusters were roughly spherical and as such, a cluster of at least 50 polyhedra would encapsulate the main structural features up to second-nearest-neighbours. In reality, most clusters had a lot more than 50 polyhedra and in fact only two of the configurations from the simulations had fewer than 100 polyhedra (Section S2†).

To determine the different structural phases formed, we calculated and compared the radial distribution functions (RDFs) of the centres of each octahedra within the clusters. Although this removed the orientational behaviour of the different phases, structures with similar RDFs were visually inspected to ensure that the orientational behaviour led to no meaningful differences between the structures. For structures at low *σ*_ang_, we were able to determine the space group of the clusters by abstracting a unit cell and using *FINDSYM*,^36,37^ a program used to identify the space group of a crystal, on a coarse-grained version of the structure and converted the solved structure back onto the equivalent structure formed with the cages. An overview of this process is given in Section S4.†

For structures at higher *σ*_ang_, as they are inherently more disordered due to the worse alignment of the cages (Fig. 3(c and d)), our procedure struggled to find a space group. In these cases, we were still able to abstract a unit cell and instead visually compared our structures to those found in the literature. An overview of which ones were solved using *FINDSYM* and which ones required visual inspection is given in Table S6† along with representative configurations of each visually solved phase (Fig. S3†). We then took the most prevalent ordered phases from the simulations and used them to colour the phase diagram by comparing the similarity of the RDF from the simulated data to the RDF of the solved phase using dynamic time warping, details of which are in Section S5.†

## 3 Results and discussion

**Fig. 4:**
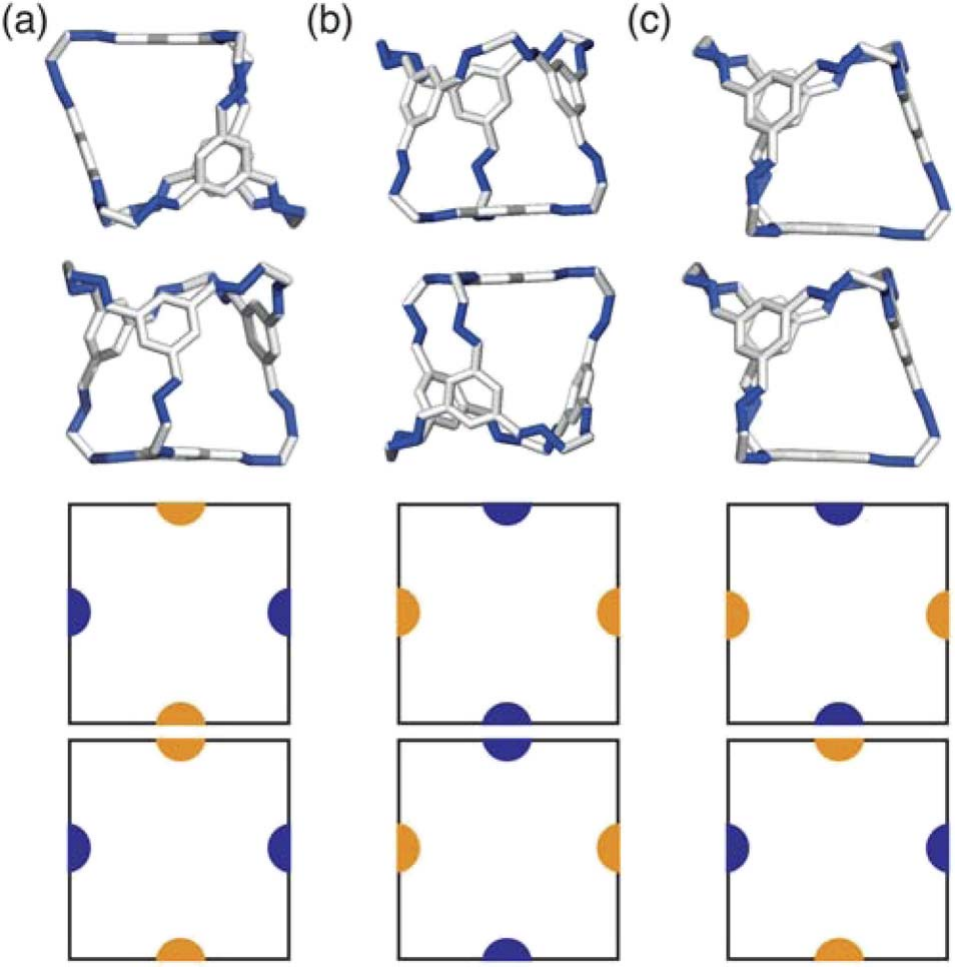
Different packing types in the simulations; (a) window-to-window, (b) arene-to-arene, and (c) window-to-arene with the corresponding favourable patchy interactions shown below in 2D. The configurations are shown with the cage CC1 for simplicity, but in reality any of the cages could be substituted into these configurations.

Due to the two different types of patches separately representing windows and arenes, we ran simulations looking at the three possible packing scenarios; (i) when interactions between only one of the types of patches is favoured *i.e.* window-to-window or arene-to-arene (Fig. 4(a and b)), (ii) when interactions between patches of the same type are favoured *i.e.* window-to-window and arene-to-arene, and (iii) when interactions between patches of different types are favoured *i.e.* window-to-arene (Fig. 4(c)). For each of these conditions, we varied the temperature and patch width, *σ*_ang_, of our model to fully explore the phase space spanned by patchy octahedra. Our results from these simulations are summarised below.

### 3.1 Phase diagrams

#### 3.1.1 Window-to-window simulations

**Fig. 5:**
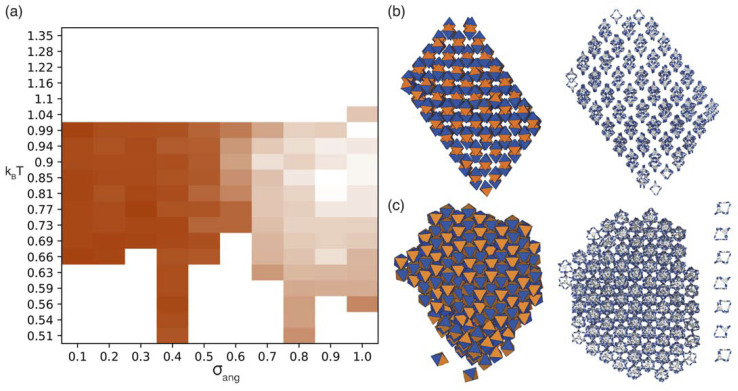
(a) Phase diagram produced from simulations where patches of one type interact with each other where the darker orange the phase is a measure of how similar the structure is to the ordered phase shown in (b). We note that uncoloured areas of the phase diagram at low temperatures are due to the simulations timing out (as explained in Section 2.3) and the phase behaviour at these temperatures is considered to be unchanged from the last simulated structure. Representative configurations of the ordered structures formed by simulations with (b) window-to-window packing and (c) window-to-window and arene-to-arene packing. Left is a representative configuration from the coarse-grained simulations, right is the mapping of the cluster onto the POC CC1 where the outer layer of the cluster has been removed. Here the different facets of the hard octahedra are coloured completely orange or blue to represent the window and arene functionality respectively. For (c) a representative chain of cages is shown on the right where there is alternating window-to-window and arene-to-arene packing.

The first simulations we performed were when only window-to-window (and by analogy arene-to-arene) packing is preferred. The low temperature RDFs only evidenced one ordered phase (Fig. S4 and S6†), a representative cluster of which is shown in Fig. 5(b) (see Fig. S7† for the analogous arene-to-arene configuration). To determine how the phase behaviour changes with temperature and also patch width, *σ*_ang_, we compared the similarities of the RDFs at each temperature and *σ*_ang_ to the RDF of the ordered phase (Fig. S6(a)†), and coloured our phase diagram (Fig. 5(a)) such that the darker orange the region of the phase diagram, the more similar our simulated structure is to the ordered phase, as described in Section S5.† The phase diagram shows that the ordered structure is present in a large area of phase space, approximately *σ*_ang_ = 0.1–0.6. At higher values of *σ*_ang_, an amorphous phase forms instead, an example of which is shown in Fig. S1(a).† When *σ*_ang_ = 1.0, although there is another phase that has an ordered RDF at low temperature (Fig. S4(j)†), on further inspection the orientation of the cages were disordered (Fig. S1(b)†). As such we deduced that this phase behaves more like a plastic crystal‡‡Although plastic crystals are typically formed by spherical particles,^24,38,39^ particle orientations in the plastic crystal phase may not be entirely random and some orientations can be more likely to occur than others,^38,40–42^ similar to what we observe in these simulations.—where the structure has positional order but orientational disorder—a phase behaviour, unsurprisingly, unreported for porous organic cages, and as such was not considered an ordered phase. Therefore, these results indicate that cages that only interact through the same type of facets (window-to-window or arene-to-arene) produce a single ordered phase in the solid state.

#### 3.1.2 Window-to-window and arene-to-arene simulations

Since the window-to-window simulations, and thus analogously the arene-to-arene simulations, only evidenced one ordered phase, to determine the phase behaviour where patches on both the windows and the arenes interact with the same patch type, we ran one targeted simulation where *σ*_ang_ = 0.2 for both the interactions representing window-to-window and arene-to-arene packing. This is equivalent to having interactions only between patches of the same type *e.g.* orange interacting with orange and blue with blue between neighbouring hard polyhedra (Fig. 3(a)). This simulation exhibited an ordered phase that contains alternating chains of window-to-window and arene-to-arene packing (Fig. 5(c)). To ensure we were not missing other potential phases by only sampling a narrow region of phase space, we sampled other combinations of *σ*_ang_ for both the interactions representing window-to-window and arene-to-arene packing between 0.1 and 0.6; these produced the same structure. The results from these simulations suggest that, similar to the window-to-window simulations, cages that interact between facets of the same type only form one ordered phase.

#### 3.1.3 Window-to-arene simulations

**Fig. 6:**
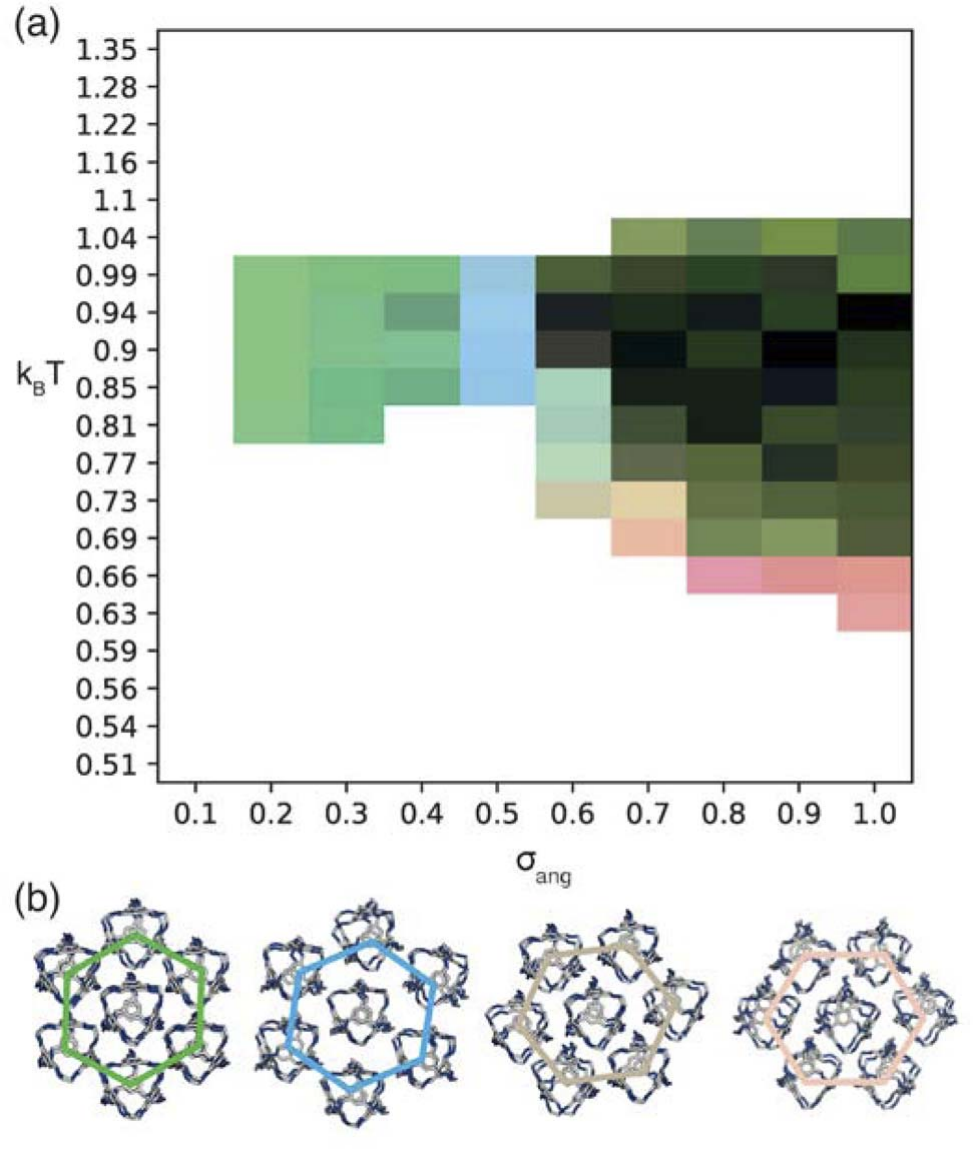
(a) Phase diagram produced from simulations where patches of opposite types interact. Here the red, green, and blue components of the phase diagram are coloured based on the similarities of the phases to low temperature structures found at *σ*_ang_ = 0.7, 0.2–0.4, and 0.5 respectively as described in more detail in Section S5.† We note that uncoloured areas of the phase diagram at low temperatures are due to the simulations timing out (as explained in Section 2.3) and the phase behaviour at these temperatures is considered to be unchanged from the last simulated structure. (b) Representative configurations of the low temperature phases showing how that angle of rotation of the nearest shell of cages rotates as a function of *σ*_ang_. From left to right the configurations represent the phases at *σ*_ang_ = 0.2–0.4, 0.5, 0.6 and 0.7. The colour of the hexagons relate to the colour of the phase diagram where the phase is found.

For simulations where patches preferentially interact with those of the opposite type, *i.e.* window-to-arene packing, we found that when *σ*_ang_ = 0.1, the configurations did not order into clusters bigger than our limit of 50 polyhedra and as such we excluded these results. For simulations where *σ*_ang_ ≥ 0.2, looking at the low temperature RDFs, we found four distinct phases which changed as a function of increasing *σ*_ang_ (Fig. S5†). We chose the three most disparate phases (in terms of their RDF, Fig. S6(b–d)†) which became the red, green, and blue components of our phase diagram, Fig. 6(a). As with the window-to-window simulations, this phase diagram loses some information as we exclude the orientational behaviour of our structure due to the RDF data containing only the central positions of the cages, but again all phases were inspected visually to ensure we were not incorrectly characterising two orientationally different structures as the same phase. As expected, the phase behaviour at low temperatures of our phase diagram evidences the four different phases seen in the RDFs, shown through the different coloured sections at *σ*_ang_ = 0.2–0.4, 0.5, 0.6, and 0.7–1.0, Fig. 6(a). To ensure we were not missing any other phases due to the discrete values of *σ*_ang_ chosen, we ran simulations at intermediate values between *σ*_ang_ = 0.4–0.7 where the majority of the different phase behaviour lies, but this did not result in any new phases.

For the four different low temperature phases, the obvious question is how the phase behaviour differs with changing *σ*_ang_. Although the RDFs at higher values of *σ*_ang_ suggest that the same phase persists from *σ*_ang_ = 0.7–1.0, on inspection of the structures, the cages orientations are disordered when *σ*_ang_ ≥ 0.8 (Fig. S8(a)†), likely due to the large values of *σ*_ang_ and *σ*_tor_ leading to less strongly directional interactions towards the facets. This means that although the phase behaviour looks similar, we are hesitant to call this the same phase and instead suggest that phases at higher *σ*_ang_ behave more like plastic crystals, much like in the case of high values of *σ*_ang_ for the window-to-window simulations.

For the four orientationally ordered phases between *σ*_ang_ = 0.2–0.7, when looking down one of the high symmetry directions, each cage is surrounded by a hexagonal arrangement of cages. Interestingly, the angle of the hexagonal arrangement around the central cage compared to the orientation of the central cage changes as a function of *σ*_ang_, Fig. 6(b). This suggests that the arrangement of the cages can be tuned almost linearly as a function of the patch width. To understand how this change in structure can affect the properties of the material, we examined the possible pore connectivity in each structure. We found that both the structures which form at small values of *σ*_ang_ may be able to host 1D pore channels due to their extrinsic porosity (Fig. S9†), whereas the two ordered structures at higher values of *σ*_ang_ can not. Therefore, understanding how the structure can be tuned as a function of patch width is a valuable task, both from a crystal engineering point of view, and for future studies focused on finding novel cages with pore connectivity. However, even if cages form either of the two structures at small values of *σ*_ang_, they might not exhibit porosity as if there were bulky groups on the vertices, the bulky groups could obstruct the connectivity by sitting within the pore channels.

On a molecular level, the manipulation of *σ*_ang_ may be achieved by changing the chemical functionality of the vertices or by using directional solvents. Bulkier groups on the vertices are likely to result in smaller values of *σ*_ang_. Although bulkier groups might be thought to change the equilibrium distance between the cages (and thus *σ*_LJ_ in eqn (1)), results from force field calculations show that there is no change in the equilibrium bond distance for cages with vastly different steric behaviour *e.g.*CC1 and CC9 as shown in Section S9.† Instead, the reason why we expect that bulkier groups can be related to a decrease in *σ*_ang_ is that the bulkier the group, the greater the energetic penalty for neighbouring cages to be unaligned from a central position—a measure of the patch width—due to repulsive steric interactions. Similarly, solvents can be used to obtain more directional interactions, for example through hydrogen bonding between the pores, and therefore smaller values of *σ*_ang_. We note that our simple model is unable to encapsulate all types of solvation effects, but instead use this as an example to highlight that some solvents may lead to more directional interactions along the 〈111〉 axes which could be used to navigate the phase behaviour seen here.

On changing temperature, the number of phase transitions seen in our simulations differs depending on the value of *σ*_ang_. At low *σ*_ang_ (0.2–0.5), each phase undergoes one phase transition at *k*_B_*T* ≈ 1 to an ordered phase that persists down to low temperature. However, when *σ*_ang_ ≥ 0.6, the structures undergo two phase transitions, one at *k*_B_*T* ≈ 1 and one at lower temperature, as evidenced by the different colours in the phase diagram (Fig. 6(a)). This first transition is to an amorphous cluster, a representative structure is included in Fig. S8(b),† and the second is to a positionally ordered phase at low temperature. Interestingly, the amorphous to ordered phase transition occurs for a wide range of *σ*_ang_, spanning areas of phase space with two distinct ordered structures at low temperature—both when *σ*_ang_ = 0.6 and 0.7—as well as the plastic crystal phases where *σ*_ang_ ≥ 0.8. The existence of two phases as a function of temperature suggests that the solid-state structure of cages which have a higher *σ*_ang_ can be controlled by temperature. Thus, cages that relate to this area of phase space may form both an amorphous and ordered structure based on the temperature of the synthesis, whereas molecules which exist at lower values of *σ*_ang_ may only evidence ordered structures no matter the synthesis temperature.

### 3.2 Comparison to known phases

**Fig. 7:**
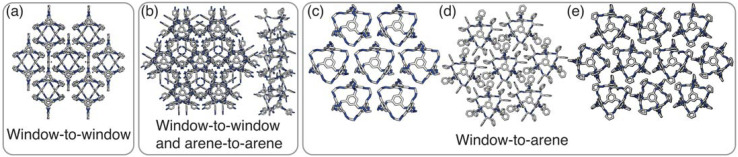
The experimentally reported enantiopure, desolvated, octahedral cages that contain no occupational disorder. (a) CC3α which is also the same reported structure for CC4β, (b) CC10, (c) CC1β′, (d) CC9-*R*3, (e) CC4α which is also the same reported structure for CC3β.

Having determined the phase behaviour observed by changing parameters of our coarse-grained model, the question remains whether it is a suitable process for predicting the phase behaviour of POCs. Given the simplicity of our model, we were particularly interested in whether we could recreate the packing behaviour spanned by enantiopure desolvated octahedral cages that do not contain any compositional disorder, of which there are five phases reported in the literature,^43^ as summarised in Table 1 and Fig. 7. The specific focus on desolvated phases is a consequence of many of the functional properties of POCs relying on the interconnected pore network which are typically disrupted in solvated phases due to solvent molecules residing within the channels. So to determine if our process had predictive capabilities, we set out to establish whether these crystal structures were found within our simulated phase diagrams.

**Table 1:** Previously reported experimental crystal structures and their dominant packing motif(s) for ordered, enantiopure, desolvated octahedral cages

Structure	Dominant packing type	Space group
PUDXES^19^ (CC3α)	Window-to-window	*F*4_1_32
OZECAY03 (ref. 44) (CC4β)		
GANDUW^45^ (CC10)	Window-to-window and arene-to-arene	*R*32
ELALAF^10^ (CC1β′)	Window-to-arene	*R*3
GANDAC^45^ (CC9-*R*3)	Window-to-arene	*R*3
PUDXES02 (ref. 44) (CC3β)	Window-to-arene	*P*3
OZECAY^46^ (CC4α)		

**Fig. 8:**
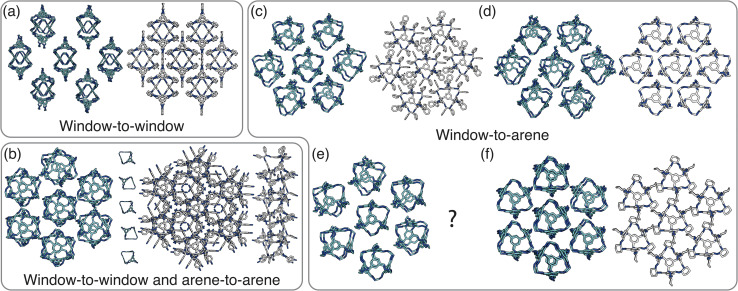
Our simulated results compared to experimental structures. A representative cluster using the cage CC1 from each simulation is shown on the left in cyan next to its analogous experimental structure on the right. (a) The ordered structure produced by window-to-window simulations which matches up to CC3α/CC4β. (b) The ordered structure produced by simulations with both window-to-window and arene-to-arene packing which matches up to the solid-state structure formed by CC10. (c–f) The ordered structures formed in window-to-arene simulations when: (c) *σ*_ang_ = 0.6 which is related to the structure formed by CC9, (d) *σ*_ang_ = 0.7 which has the same structure as CC1β′, (e) *σ*_ang_ = 0.5 which is unreported in the literature, and (f) *σ*_ang_ = 0.2–0.4, which is related to the solvated form of CC3 with Et_2_O and CH_2_Cl_2_ and CC4 with MeOH.

For cages where the dominant packing type is window-to-window, and a combination of window-to-window and arene-to-arene, only one solid-state structure is reported to exist (Fig. 7(a), window-to-window packing, and Fig. 7(b), window-to-window and arene-to-arene packing). This observation fits well with our simulations which also produce only one ordered structure from the two simulations, and on further analysis the structures produced in the simulations correspond to those seen experimentally (Fig. 8(a and b)).

When the dominant packing type is window-to-arene, three different crystal structures are reported to exist experimentally (Fig. 7(c–e)) which seemingly corroborates our window-to-arene results from the simulations that evidence a much richer phase diagram than for window-to-window. Yet, only two of the three experimentally known phases are found in our phase diagram, CC9-*R*3 (at *σ*_ang_ = 0.6) (Fig. 8(c)) and CC1β′ (at *σ*_ang_ = 0.7) (Fig. 8(d)). The remaining phase reported experimentally does not exist within any of our phase diagrams. However, this is not a surprising result. Experimentally, this structure is formed with both cages CC3 and CC4 upon desolvation from another ordered structure. As such, it has been suggested that the crystal structure identified is formed due to a solvent templation effect resulting in a single-crystal-to-single-crystal transformation upon desolvation, something not taken into account by our model, and so it is not expected to be found in our simulations.^44,46^ This result highlights how solvent can be used to alter the polymorphic behaviour of the crystal and even change the preference of the dominant interaction between facets. In this case, the change in packing behaviour for CC3 from CC3α to CC3β would likely decrease the porosity from the loss of the three dimensional pore network, however, for CC4, *para*-xylene can direct the packing behaviour from window-to-arene to window-to-window as seen in the formation of CC4β.^44^

While our results show that we can predict most of the crystal structures known to be formed by ordered, enantiopure, desolvated octahedral cages, there still remains two ordered phases produced in our window-to-arene simulations that do not match up to any of the structures in Table 1. The first of these structures is a novel phase (when *σ*_ang_ = 0.5) (Fig. 8(e)) not reported within the literature, and the second is found in solvated forms of both CC3 with Et_2_O and CH_2_Cl_2_ and CC4 with MeOH (Fig. 8(f)). Although we were not intending to find solvated structures, the occurrence of the solvated crystal in this region of phase space is logical as the structure occurs at low values of *σ*_ang_ = 0.2–0.4, which suggests the inclusion of the solvent increases the directionality, as it occurs at small patch widths. This is a logical result as the inclusion of solvents is likely to make the intermolecular interactions more directional, analogous to decreasing *σ*_ang_. Moreover, this phase is the same as the solvated structure which produces CC3β/CC4α upon desolvation.^44,46^ We note that the discussion of whether the affects of solvent can be represented by our model depends on how the solvent affects the phases. When the solvent is included in the pores, it leads to a more directional interaction which is able to be captured by our model. However, when the solvent is removed from the pores, as in the case for CC3β/CC4α, the act of desolvation disrupts the directional interactions introduced by the solvent without allowing the phase to properly re-equilibrate, causing a phase transition which our model cannot capture.^44,46^ An overview of where each experimental phase is found in our simulations is give in Table 2.

**Table 2:** Match between the parameters of the coarse-grained model that produce different crystal structures and their related experimentally reported structures

Experimentally reported structure	Dominant packing type	*σ*_ang_
PUDXES (CC3α)	Window-to-window	0.1–0.6
OZECAY03 (CC4β)		
GANDUW (CC10)	Window-to-window/arene-to-arene	0.1–0.6/0.1–0.6
NODVIN (CC3 with Et_2_O and CH_2_Cl_2_)	Window-to-arene	0.2–0.4
OZEBUR (CC4 with MeOH)		
N/A	Window-to-arene	0.5
GANDAC (**CC9**-*R*3)	Window-to-arene	0.6
ELALAF (**CC1β′**)	Window-to-arene	0.7
PUDXES02 (**CC3β**)	Window-to-arene	N/A
OZECAY (**CC4α**)		

We have shown that when window-to-window packing dominates, only one phase is formed, as seen experimentally, whereas window-to-arene packing results in a much richer phase space containing four phases, only three of which have been seen experimentally. The coarse-grained model laid out in eqn (1) is able to produce the majority of the structures formed by the enantiopure, ordered, desolvated octahedral cages. By changing the width of the patch, and thus the directionality of the interactions between cages, we are able to manipulate the structural phases formed by our model, mimicking the structural changes seen when changing the chemical functionality. Now, the natural question is, given a novel cage, can we predict its likely solid-state structure using our simulations? And what design rules can we assimilate from our coarse-grained model?

### 3.3 Predictive capabilities and design rules

In order to gain insight into the most dominant packing types (*e.g.* window-to-arene) and likely value of *σ*_ang_, a process must be created to relate the interactions between cages, as calculated through atomistic methods, to our phase space. Although density functional theory (DFT) dimer pair calculations examining packing motifs have proven to be deterministic of preferred packing behaviour,^10^ there is no obvious reason why one cage prefers one packing type over the other. This subtlety is what makes the crystal engineering of POCs so difficult. Moreover, establishing a robust relationship between DFT results and a continuous parameter such as *σ*_ang_ is a more complex task. We have started to address this problem by examining the energetics of two cages slipping around the central patch position (see Section S9†). The goal here is to relate the energies produced from atomistic calculations to the parameters of our model, creating a relationship between each region of our phase space to a range of interaction energies. Therefore, by using this relationship for novel cages, we can use atomistic simulations of two cage molecules to extrapolate to solid-state, crystalline phase behaviour. However, for both the window-to-window and window-to-arene simulations there are only two desolvated cages that have been reported to form any of the predicted crystal structures: (i) CC9-*R*3 and CC1β′ in the window-to-arene simulations, (ii) CC3α and CC4β in the window-to-window simulations. Moreover, the predicted crystal structures are found in a narrow, if not the same, region of phase space. This means that we are unable to relate the energies produced from atomistic calculations to the parameters of our model and thus crystal phase behaviour.

Nevertheless, for the cages which pack preferentially *via* their window and arene facets, the energetic differences produced by the two cages slipping around the central patch position are in agreement with where their crystal structures are found relative to one another in the phase diagram. CC9 has a higher energetic penalty associated with displacements from the central patch position than CC1, mirroring the relationship that the solid-state structure of CC9-*R*3 is found at a lower *σ*_ang_ than CC1β′ (Fig. S12†). These results indicate that similar calculations looking at the slipping of cages might be able to predict the packing behaviour of novel cages, particularly once a threshold is established between atomistic results and *σ*_ang_. For this we will need to find molecular cages whose crystalline structures relate to areas of our phase space currently unobserved experimentally.

As for design rules, our results have started to establish a relationship between chemical functionality, solvent, and solid-state phase space as represented by our model. Cages synthesised with bulkier diamine vertices (*e.g.*CC3, CC9, and CC10) tend to sit at lower values of *σ*_ang_ than less bulky cages such as CC1 (Table 2). Additionally, the inclusion of solvent, as in the case of structures formed by CC3 and CC4, leads to low values of *σ*_ang_. These trends can inform design rules, as they show that solvating groups can be used to introduce more directional interactions, increasing the number of polymorphs available for a given cage and thus opportunities for increased porosity, and that bulkier groups on the vertices lead to less rotation and slipping between neighbouring cages.

Finding more desolvated cages whose solid-state structures match those in our phase space, particularly for structures at low *σ*_ang_, will lead to the development of a better relationship between results from atomistic calculations and the parameters of our coarse-grained model. This can lead to more concrete rules about the relative energies required to navigate phase space, allowing for easy prediction of the crystal structures formed by novel cages in a much less computationally expensive process than for CSP—only requiring dimer pair DFT calculations compared to many solid-state structure calculations. Through this we hope to further decipher the relationship between solvent, chemical functionality, and *σ*_ang_ to indicate design rules for tuning the solid-state phase behaviour of POCs.

On top of information on the structural behaviour of cages, our results may hold insights on the crystallisation process of POCs. The window-to-arene simulations show that, depending on the temperature of the simulation, either an amorphous or ordered phase forms. Perhaps a similar trend could be found with synthesis temperatures, forming a link between our simulations and crystallisation effects such that our model can act as a guide for POCs crystallisation pathways. For example, our phase diagram suggests that crystallising CC9 or CC1 at high temperatures could lead to the formation of amorphous phases, whereas at low temperature an ordered crystalline structure will form. However for cages such as CC3, or any cages that pack window-to-window, the phase formed is independent of synthesis temperature as either an ordered phase forms at any temperature, as in CC3, or an amorphous phase is formed, as with cages that exist at higher *σ*_ang_. This is a particularly useful result as it highlights routes for targeting amorphous cages which can exhibit enhanced gas selectivity^47^ and increased porosity relative to their crystalline counterparts.^47–49^ Moreover, our results may provide insight into the polymorphic behaviour of POCs by giving an overview of how many phases exist given the dominant interaction type. For example, for window-to-window packing, the only known ordered structure is that of the 3D connected diamondoid structure found in CC3, which matches our window-to-window simulation results, whereas the window-to-arene simulations exhibit a much richer polymorphic phase space, as seen experimentally.

## 4 Conclusion

This work presents a coarse-grained model to examine the solid-state packing behaviour of POCs with dominant window-to-window and window-to-arene packing. Our results show that when cages interact most strongly through their windows then only one phase is seen. However, when cages interact most strongly between the windows and arenes, a much richer phase diagram is produced with four structurally distinct phases. These observations match up well to experimental results, where only one solid-state structure is reported when window-to-window packing dominates, but a range of phases are observed for window-to-arene packing.

Through these simulations, we have shown that our coarse-grained model is able to reproduce the majority of the packing behaviour seen by the enantiopure, ordered, desolvated octahedral cages. These results are the first steps towards using computationally inexpensive coarse-grained simulations to predict the packing of POCs from their molecular structure, as well as developing design rules based on the chemical functionality of the vertices. Here we have shown how both solvent and the size of the functional group on the cages vertices can be used to direct crystal structure formation through changing the directionality of the interactions. Thus, both the addition of solvent and the inclusion of bulky functional groups can manipulate structural formation by reducing rotation and slipping between neighbouring cages, changing the packing behaviour. Additionally we have shown that synthesis temperature may be used to influence the crystallinity in cages, guiding efforts for amorphous phase formation which can lead to increased porosity.^48^ As far as we are aware, this is the first study that looks to coarse-grain molecules for crystal structure prediction. We hope that the results highlighted here will be expanded upon to use coarse-grained modelling for molecules with a richer experimental phase space in order to map molecular interactions onto coarse-grained phase space using processes outlined in Section 3.3 for novel molecular structure prediction.

Although the focus of this paper has been on POCs, the results outlined here have ramifications in many areas of molecular chemistry. Motifs common in POCs are also widespread in other porous materials such as elemental carbon cages^50^ and metal–organic cages.^31^ Therefore, solid-state structures predicted by methods outlined in this study may also relate to crystals formed by other molecular systems and the methodology outlined in this paper could be expanded to other areas of molecular chemistry to help predict the organic solid-state. Eventually, we aim to introduce our computationally inexpensive methodology into existing high-throughput workflows using robotic automation and computational modelling,^15^ creating a streamlined workflow for structural prediction of novel molecules. Applying this process to multi-component systems could help drive phase exploration at orders of magnitude currently intractable by current computational techniques.

## Data availability

The structures of novel, amorphous, and plastic phases simulated here have been uploaded as part of the ESI.†

## Author contributions

E. H. W. conceptualised and designed the project, carried out and analysed the simulations. K. E. J. supervised the project and acquired funding. E. H. W. wrote the manuscript and all authors contributed to the final version.

## Conflicts of interest

There are no conflicts to declare.

## Supplementary Material

SC-013-D2SC04511G-s001

SC-013-D2SC04511G-s002

SC-013-D2SC04511G-s003

SC-013-D2SC04511G-s004

SC-013-D2SC04511G-s005

SC-013-D2SC04511G-s006

SC-013-D2SC04511G-s007

## References

[cit1] Ostroverkhova O. (2016). Chem. Rev..

[cit2] Cooper A. I. (2017). ACS Cent. Sci..

[cit3] Datta S., Grant D. J. W. (2004). Nat. Rev. Drug Discovery.

[cit4] Zhao C., Chen L., Che Y., Pang Z., Wu X., Lu Y., Liu H., Day G. M., Cooper A. I. (2021). Nat. Commun..

[cit5] Pyzer-Knapp E. O., Thompson H. P. G., Schiffmann F., Jelfs K. E., Chong S. Y., Little M. A., Cooper A. I., Day G. M. (2014). Chem. Sci..

[cit6] Case D. H., Campbell J. E., Bygrave P. J., Day G. M. (2016). J. Chem. Theory Comput..

[cit7] McMahon D. P., Stephenson A., Chong S. Y., Little M. A., Jones J. T. A., Cooper A. I., Day G. M. (2018). Faraday Discuss..

[cit8] Reilly A. M., Cooper R. I., Adjiman C. S., Bhattacharya S., Boese A. D., Brandenburg J. G., Bygrave P. J., Bylsma R., Campbell J. E., Car R., Case D. H., Chadha R., Cole J. C., Cosburn K., Cuppen H. M., Curtis F., Day G. M., DiStasio Jr R. A., Dzyabchenko A., van Eijck B. P., Elking D. M., van den Ende J. A., Facelli J. C., Ferraro M. B., Fusti-Molnar L., Gatsiou C.-A., Gee T. S., de Gelder R., Ghiringhelli L. M., Goto H., Grimme S., Guo R., Hofmann D. W. M., Hoja J., Hylton R. K., Iuzzolino L., Jankiewicz W., de Jong D. T., Kendrick J., de Klerk N. J. J., Ko H.-Y., Kuleshova L. N., Li X., Lohani S., Leusen F. J. J., Lund A. M., Lv J., Ma Y., Marom N., Masunov A. E., McCabe P., McMahon D. P., Meekes H., Metz M. P., Misquitta A. J., Mohamed S., Monserrat B., Needs R. J., Neumann M. A., Nyman J., Obata S., Oberhofer H., Oganov A. R., Orendt A. M., Pagola G. I., Pantelides C. C., Pickard C. J., Podeszwa R., Price L. S., Price S. L., Pulido A., Read M. G., Reuter K., Schneider E., Schober C., Shields G. P., Singh P., Sugden I. J., Szalewicz K., Taylor C. R., Tkatchenko A., Tuckerman M. E., Vacarro F., Vasileiadis M., Vazquez-Mayagoitia A., Vogt L., Wang Y., Watson R. E., de Wijs G. A., Yang J., Zhu Q., Groom C. R. (2016). Acta Crystallogr., Sect. B: Struct. Sci., Cryst. Eng. Mater..

[cit9] Hasell T., Chong S. Y., Schmidtmann M., Adams D. J., Cooper A. I. (2012). Angew. Chem., Int. Ed..

[cit10] Jones J. T. A., Hasell T., Wu X., Bacsa J., Jelfs K. E., Schmidtmann M., Chong S. Y., Adams D. J., Trewin A., Schiffman F., Cora F., Slater B., Steiner A., Day G. M., Cooper A. I. (2011). Nature.

[cit11] Slater A. G., Cooper A. I. (2015). Science.

[cit12] Brutschy M., Schneider M. W., Mastalerz M., Waldvogel S. R. (2012). Adv. Mater..

[cit13] Yang X., Sun J.-K., Kitta M., Pang H., Xu Q. (2018). Nat. Catal..

[cit14] Hasell T., Cooper A. I. (2016). Nat. Rev. Mater..

[cit15] Greenaway R. L., Santolini V., Bennison M. J., Alston B. M., Pugh C. J., Little M. A., Miklitz M., Eden-Rump E. G. B., Clowes R., Shakil A., Cuthbertson H. J., Armstrong H., Briggs M. E., Jelfs K. E., Cooper A. I. (2018). Nat. Commun..

[cit16] Bennett S., Szczypiński F. T., Turcani L., Briggs M. E., Greenaway R. L., Jelfs K. E. (2021). J. Chem. Inf. Model..

[cit17] Greenaway R. L., Jelfs K. E. (2021). Adv. Mater..

[cit18] He A., Jiang Z., Wu Y., Hussain H., Rawle J., Briggs M. E., Little M. A., Livingston A. G., Cooper A. I. (2022). Nat. Mater..

[cit19] Tozawa T., Jones J. T. A., Swamy S. I., Jiang S., Adams D. J., Shakespeare S., Clowes R., Bradshaw D., Hasell T., Chong S. Y., Tang C., Thompson S., Parker J., Trewin A., Bacsa J., Slawin A. M. Z., Steiner A., Cooper A. I. (2009). Nat. Mater..

[cit20] Hasell T., Culshaw J. L., Chong S. Y., Schmidtmann M., Little M. A., Jelfs K. E., Pyzer-Knapp E. O., Shepherd H., Adams D. J., Day G. M., Cooper A. I. (2014). J. Am. Chem. Soc..

[cit21] Desiraju G. R. (2013). J. Am. Chem. Soc..

[cit22] Anderson J. A., Glaser J., Glotzer S. C. (2020). Comput. Mater. Sci..

[cit23] Cadotte A. T., Dshemuchadse J., Damasceno P. F., Newman R. S., Glotzer S. C. (2016). Soft Matter.

[cit24] Damasceno P. F., Engel M., Glotzer S. C. (2012). Science.

[cit25] Harper E. S., van Anders G., Glotzer S. C. (2019). Proc. Natl. Acad. Sci. U. S. A..

[cit26] van Anders G., Klotsa D., Ahmed N. K., Engel M., Glotzer S. C. (2014). Proc. Natl. Acad. Sci. U. S. A..

[cit27] Nguyen T. D., Phillips C. L., Anderson J. A., Glotzer S. C. (2011). Comput. Phys. Commun..

[cit28] Mansbach R. A., Ferguson A. L. (2018). J. Phys. Chem. B.

[cit29] Long A. W., Ferguson A. L. (2018). Mol. Syst. Des. Eng..

[cit30] Groom C. R., Bruno I. J., Lightfoot M. P., Ward S. C. (2016). Acta Crystallogr., Sect. B: Struct. Sci., Cryst. Eng. Mater..

[cit31] Young N. J., Hay B. P. (2013). Chem. Commun..

[cit32] Santolini V., Miklitz M., Berardo E., Jelfs K. E. (2017). Nanoscale.

[cit33] Anderson J. A., Eric Irrgang M., Glotzer S. C. (2016). Comput. Phys. Commun..

[cit34] Noya E. G., Wong C. K., Llombart P., Doye J. P. K. (2021). Nature.

[cit35] Wilber A. W., Doye J. P. K., Louis A. A., Lewis A. C. F. (2009). J. Chem. Phys..

[cit36] Stokes H. T., Hatch D. M. (2005). J. Appl. Crystallogr..

[cit37] Stokes H. T. , Hatch D. M. and Campbell B. J., FINDSYM, ISOTROPY Software Suite, https://iso.byu.edu

[cit38] Gantapara A. P., de Graaf J., van Roij R., Dijkstra M. (2015). J. Chem. Phys..

[cit39] Agarwal U., Escobedo F. A. (2011). Nat. Mater..

[cit40] Sharma A. K., Thapar V., Escobedo F. A. (2018). Soft Matter.

[cit41] Burian M., Karner C., Yarema M., Heiss W., Amenitsch H., Dellago C., Lechner R. T. (2018). Adv. Mater..

[cit42] Shen W., Antonaglia J., Anderson J. A., Engel M., van Anders G., Glotzer S. C. (2019). Soft Matter.

[cit43] Bernabei M., Pérez-Soto R., Gómez García I., Haranczyk M. (2018). Mol. Syst. Des. Eng..

[cit44] Little M. A., Chong S. Y., Schmidtmann M., Hasell T., Cooper A. I. (2014). Chem. Commun..

[cit45] Bojdys M. J., Briggs M. E., Jones J. T. A., Adams D. J., Chong S. Y., Schmidtmann M., Cooper A. I. (2011). J. Am. Chem. Soc..

[cit46] Mitra T., Wu X., Clowes R., Jones J. T. A., Jelfs K. E., Adams D. J., Trewin A., Bacsa J., Steiner A., Cooper A. I. (2011). Chem.–Eur. J..

[cit47] Jiang S., Jelfs K. E., Holden D., Hasell T., Chong S. Y., Haranczyk M., Trewin A., Cooper A. I. (2013). J. Am. Chem. Soc..

[cit48] Evans J. D., Huang D. M., Hill M. R., Sumby C. J., Sholl D. S., Thornton A. W., Doonan C. J. (2015). J. Phys. Chem. C.

[cit49] Hasell T., Chong S. Y., Jelfs K. E., Adams D. J., Cooper A. I. (2012). J. Am. Chem. Soc..

[cit50] Maier G. (1988). Angew. Chem., Int. Ed. Engl..

