# Supplementary material for: Coarse-grained modelling to predict the packing of porous organic cages
Source: Chem Sci. 2022 Oct 25;13(45):13588–99. doi: 10.1039/d2sc04511g (PMC9683088; doi:10.1039/d2sc04511g)
Supplement: SC-013-D2SC04511G-s002 [file SC-013-D2SC04511G-s002.pdf]

Electronic Supplementary Material (ESI) for Chemical Science.
This journal is © The Royal Society of Chemistry 2022

Supporting Information:

Coarse-grained modelling to predict the packing of porous organic cages

Emma H. Wolpert* and Kim E. Jelfs*

Department of Chemistry, Imperial College London, Molecular Sciences Research Hub, White City Campus, Wood Lane, London, W12 0BZ, UK

E-mail: e.wolpert@imperial.ac.uk,k.jelfs@imperial.ac.uk

Contents

1 Parameters for the coarse-grained model S-3

2 Sizes of clusters for phase determination S-4

3 Additional structural images S-5

4 Structural determination S-6

5 Phase diagram determination S-10

6 Arene-to-arene configuration S-14

7 High σ_{ang} leads to disorder S-15

8 Porous window-to-arene configurations S-16

9 Predictive capabilities S-17

References S-22

1 Parameters for the coarse-grained model

Table S1: Interaction parameters used in the window-to-window simulations.

σ_{ang}	J (kJmol^{-1})
0.1	96
0.2	56
0.3	46
0.4	41
0.5	36
0.6	30
0.7	25
0.8	21
0.9	18
1.0	17

Table S2: Interaction parameters used in the window-to-arene simulations.

σ_{ang}	J (kJmol^{-1})
0.1	-
0.2	73
0.3	50
0.4	38
0.5	28
0.6	19
0.7	15
0.8	12
0.9	10
1.0	9

Table S3: Values of the offset value for the torsional modulation term used in each simulation.

Packing type	Offset angle
window-to-window	$\frac{\pi}{2}$
window-to-arene	0

2 Sizes of clusters for phase determination

Table S4: Cluster sizes for phase determination in the window-to-window simulations.

	σ_{ang}									
k_BT	0.1	0.2	0.3	0.4	0.5	0.6	0.7	0.8	0.9	1.0
1.04	-	-	-	-	-	-	-	-	-	117
0.99	139	391	397	399	354	159	218	100	142	257
0.94	365	407	433	429	412	288	292	247	201	270
0.90	224	433	439	437	417	376	344	300	304	330
0.85	414	435	441	444	439	394	395	383	332	357
0.81	453	442	437	447	419	410	396	406	392	373
0.77	222	438	441	453	415	411	423	407	406	410
0.73	232	432	427	441	409	417	428	424	415	429
0.69	231	433	429	436	405	-	428	443	420	418
0.66	235	439	-	424	402	-	433	415	422	417
0.63	-	-	-	431	-	-	436	434	430	419
0.59	-	-	-	427	-	-	-	449	425	422
0.56	-	-	-	441	-	-	-	426	-	424
0.54	-	-	-	440	-	-	-	430	-	-
0.51	-	-	-	441	-	-	-	431	-	-

We note that due to the very directional interactions of the patches in the 0.1 simulations, two clusters had formed. Thoroughout the simulation these clusters were constantly combining and breaking apart from each other, leading to the variation in the cluster size.

Table S5: Cluster sizes for phase determination in the window-to-arene simulations.

	σ_{ang}									
k_BT	0.1	0.2	0.3	0.4	0.5	0.6	0.7	0.8	0.9	1.0
1.04	-	-	-	-	-	-	103	81	61	109
0.99	-	434	436	424	437	182	245	226	188	175
0.94	-	449	444	432	448	291	332	314	311	319
0.90	-	448	439	430	440	337	356	351	338	283
0.85	-	443	439	423	448	427	404	380	372	380
0.81	-	445	442	-	-	436	421	401	378	386
0.77	-	444	-	-	-	440	422	420	417	422
0.73	-	-	-	-	-	441	438	419	409	417
0.69	-	-	-	-	-	-	433	426	426	425
0.66	-	-	-	-	-	-	-	446	427	430
0.63	-	-	-	-	-	-	-	-	-	436
0.59	-	-	-	-	-	-	-	-	-	-
0.56	-	-	-	-	-	-	-	-	-	-
0.54	-	-	-	-	-	-	-	-	-	-
0.51	-	-	-	-	-	-	-	-	-	-

3 Additional structural images

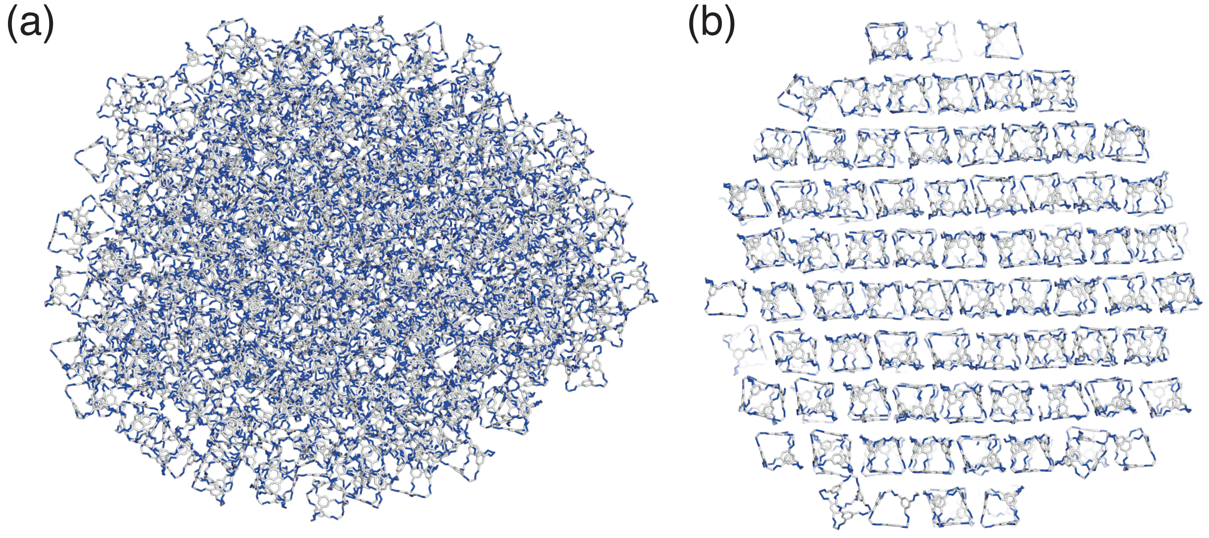

Figure S1: (a) Representative cluster of the amorphous phase formed in window-to-window simulations when $\sigma_{ang} \geq 0.7$. Here the cluster is taken from the low temperature simulation when $\sigma_{ang} = 0.9$. (b) Cross section of the plastic phase formed at low temperatures when $\sigma_{ang} = 1.0$, evidencing orientational disorder. CIF files for both these phases are provided in the ESI.

4 Structural determination

Clusters formed from HOOMD-Blue simulations were transformed onto clusters of cages using custom made code. This code transformed for the distance between neighbouring octahedra from ≈ 0.6 Å as in the simulations, to ≈ 20 Å between the cages for easy visual examination of the structure. A second custom made code then picked out a unit cell of repeating cages (Fig. S2(a)). As the unit cell contained orientations of the cages which were slightly disordered due to the nature of the simulations, the structure was coarse-grained in order to use traditional symmetry solving algorithms. The coarse graining process worked by creating a structure with only the central position of the cage and the position of three of the carbons of the arene, in order to preserve the symmetry of the cage (Fig. S2(a-c)). Using this coarse-grained structure, *FINDSYM*[S1,S2] was employed to determine the structures space group. The same transformation as invoked by *FINDSYM* to the coarse-grained unit cell, was then applied to the original unit cell (Fig. S2(e)). Using the solved space group, the symmetry conditions were then applied to the unit cell, and the sites that

related to the same atomic position were merged to get the full structure of the cage under symmetry conditions. This process worked best for cages with a small patch width, but for some of the ordered phases at large patch widths for the window-to-arene simulations, the orientation of the coarse-grained structures were still too disordered to solve using this process, *i.e.* *FINDSYMM* was unable to find an ordered structure even with the coarse-grained configuration. In these cases, the clusters were instead compared visually to known solid-state structures of octahedral porous organic cages. An overview of which of the space groups of the window-to-arene structures were solved using the coarse-graining process and which were solved visually is given in Table S6.

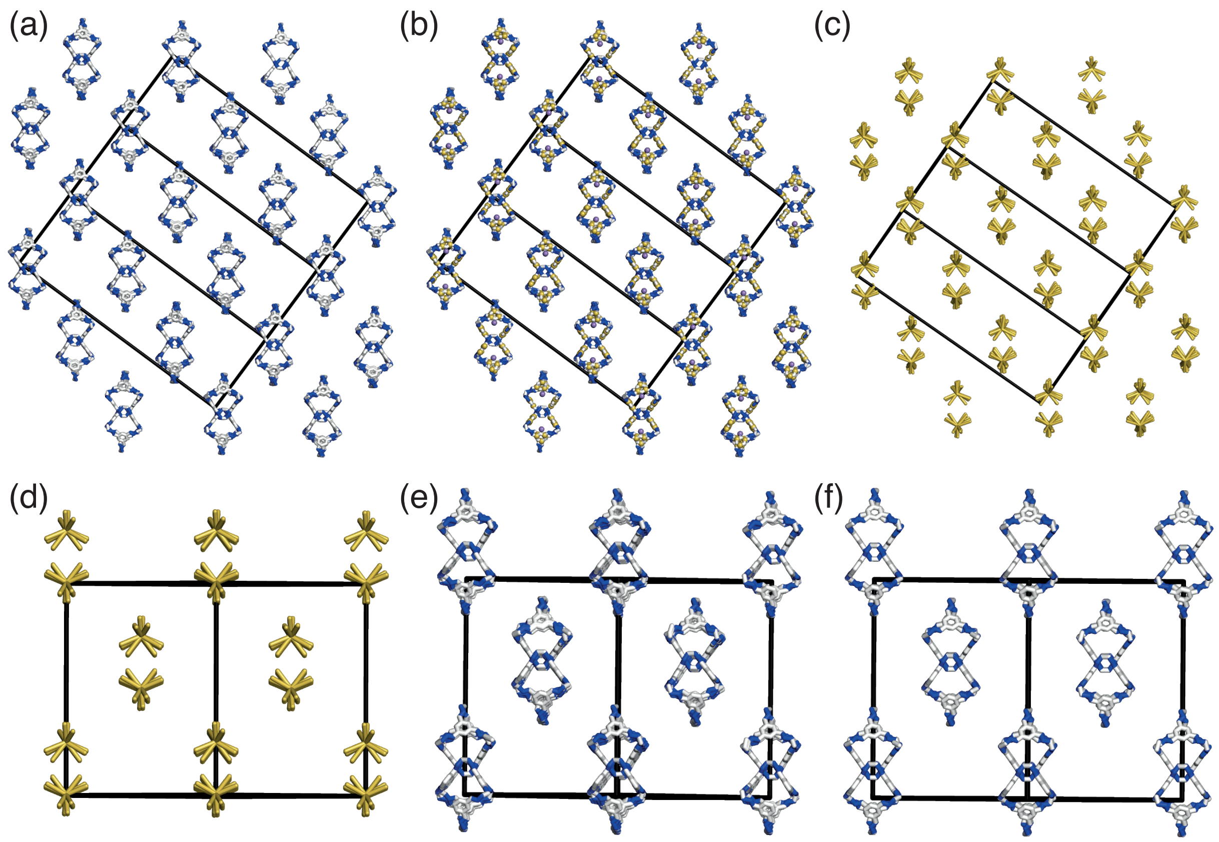

Figure S2: Overview of the process used for finding the space group of the structures shown for the ordered window-to-window simulation when $\sigma_{ang} = 0.3$. (a) An example unit cell (shown in black) picked out from the simulated cluster with the positions of the cages neatened to be on likely high symmetry sites *i.e.* $\frac{1}{4}, \frac{2}{4}, \frac{3}{4}$ or $\frac{1}{3}, \frac{2}{3}$ *etc.* (b) Addition of a central atom to each cage (purple) and selection of three of the carbon atoms (yellow) on the arene chosen to preserve the symmetry of the cage whilst reducing the degrees of freedom. (c) The structure of just the central position and three arene carbon atoms used in *FINDSYM* to get the space group of the structure. (d) The outputted structure from *FINDSYM* with the space group $F4_132$. (e) Structure obtained by converting the original unit cell to the same unit cell as outputted from *FINDSYM* with no symmetry operations. (f) Final structure obtained by applying the symmetry conditions of the space group onto the structure shown in (e) and merging the atoms that sit around the same crystallographic sites.

Table S6: Whether the structure was solved by eye or by findsym

σ_{ang}	Solved using coarse-graining and findsym
0.2-0.4	Yes
0.5	Yes
0.6	No
0.7	No
0.8-1.0	NA

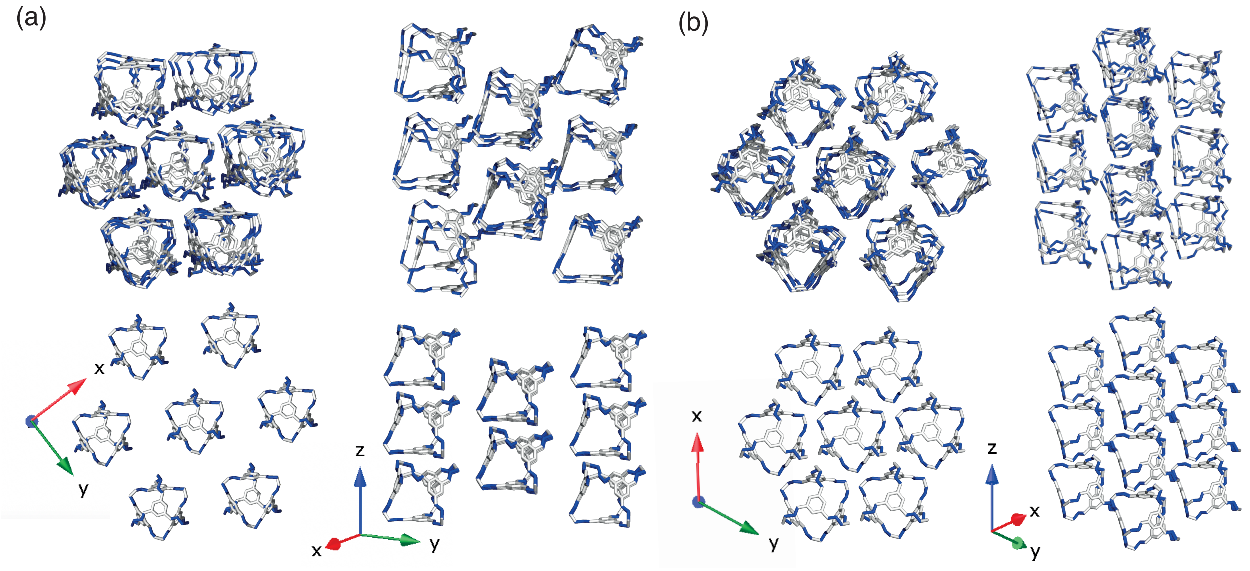

Figure S3: Comparison of the simulated phases of the window-to-arene simulations (top) to their corresponding experimental phases (bottom) as solved visually. (a) $\sigma_{ang} = 0.6/\mathbf{CC9}$, the experimental structure is derived from CC9 with the vertex functional groups (phenyl groups) removed for easy comparison. (b) $\sigma_{ang} = 0.7/\mathbf{CC1}\beta$.

5 Phase diagram determination

The phase diagrams for both the window-to-window and window-to-arene simulations were coloured using a continuous colour map based on their similarities to the RDF of the solved ordered structures at low temperature Fig. S6. For phases at small patch widths, the structures are relatively ordered and as such the RDFs were compared to the RDFs of the ordered structure calculated with 40 bins, *i.e.* a high resolution RDF (Fig. S6(a),(c)). On the other hand, the phases at large σ_{ang} are inherently more disordered, and as such so are their RDFs Fig. S4,S5. Therefore, the similarities between the simulated and solved structure was compared using an RDF with a fewer number of bins (25) *i.e.* an RDF lower resolution. The comparisons between the RDFs were made by using a time series analysis, dynamic time warping, an algorithm commonly used for measuring the similarity between two temporal sequences. This analysis has been used to compare the difference between two PXRDs before.[S3] In this study, we used it to compare RDFs by first normalising the RDFs such that the largest value of $g(r) = 1$, and then calculating the dynamic time warping. The values returned by the algorithim were then normalised for both the window-to-window and window-to-arene simulation, which gave a similarity measure between 0-1 for each ordered RDF where 1 is the most similar and 0 the least. This similarity measure was then used to colour the phase diragrams. For the window-to-window simulation, given there was only one phase deemed to have both orientational and translational order, the phase diagram was coloured orange by the similarity measure to said phase (Fig. S6(a)). For the window-to-arene simulation, as there were four separate ordered phases, the similarites of the RDFs for the most disparate three were used to colour the phase diagram red, green, and blue (Fig. S6(b-d)).

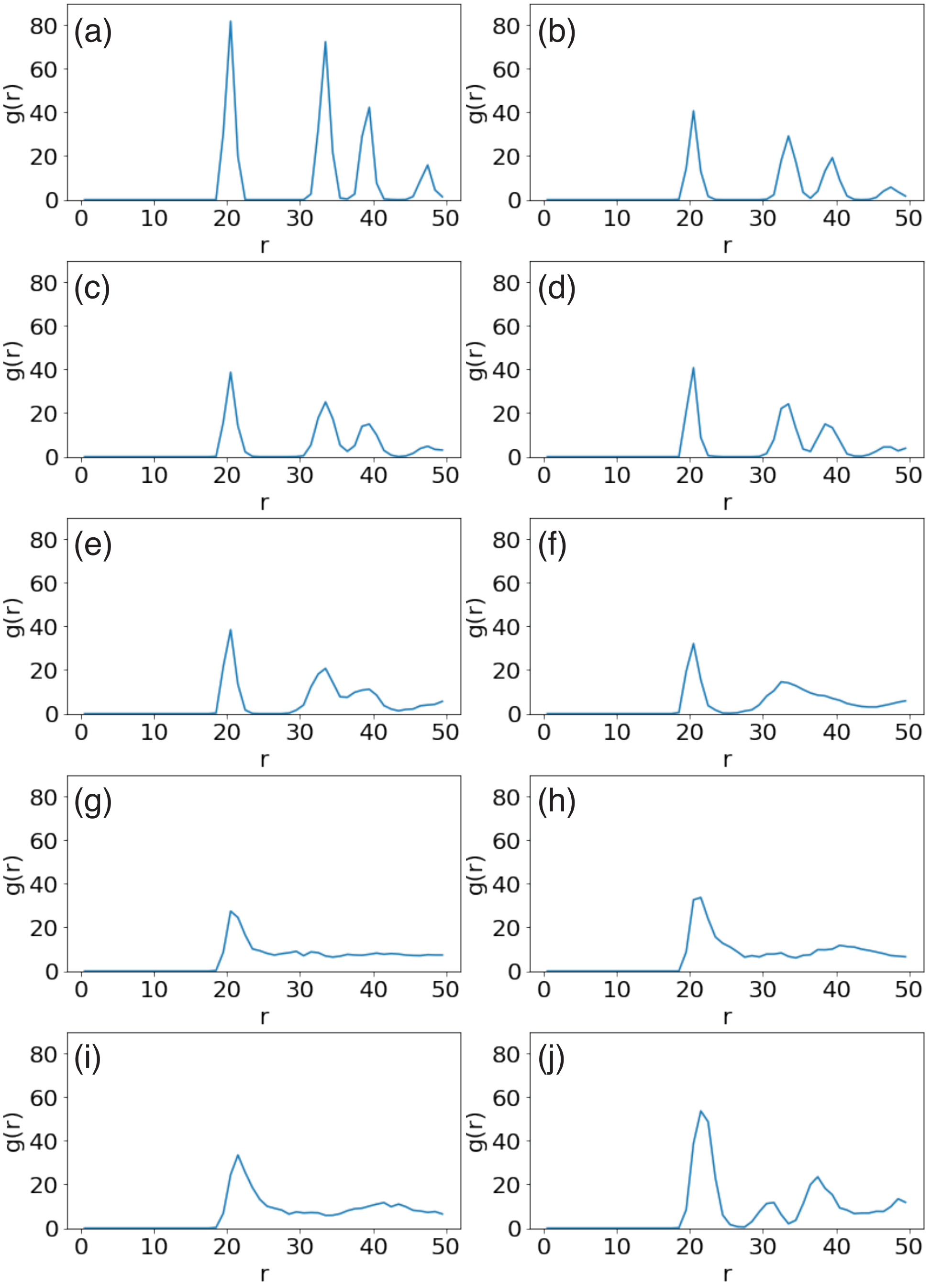

Figure S4: The RDFs for the low temperature structures from the window-to-window simulations when (a) $\sigma_{ang} = 0.1$, (b) 0.2, (c) 0.3, (c) 0.4, (d) 0.5, (e) 0.6, (f) 0.7, (g) 0.8, (h) 0.9, and (i) 1.0.

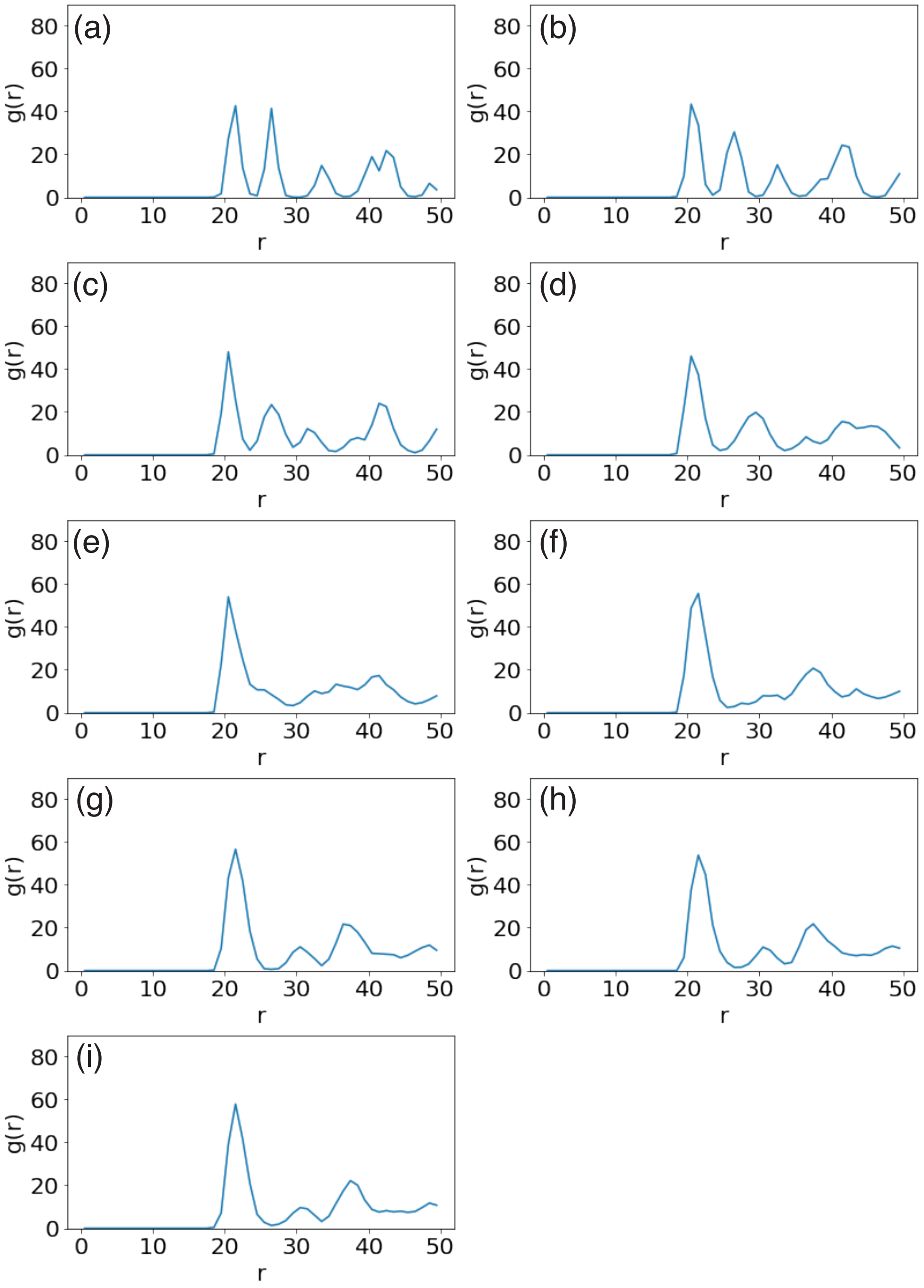

Figure S5: The RDFs for the low temperature structures from the window-to-window simulations when (a) $\sigma_{ang} = 0.2$, (b) 0.3, (c) 0.4, (c) 0.5, (d) 0.6, (e) 0.7, (f) 0.8, (g) 0.9, and (h) 1.0.

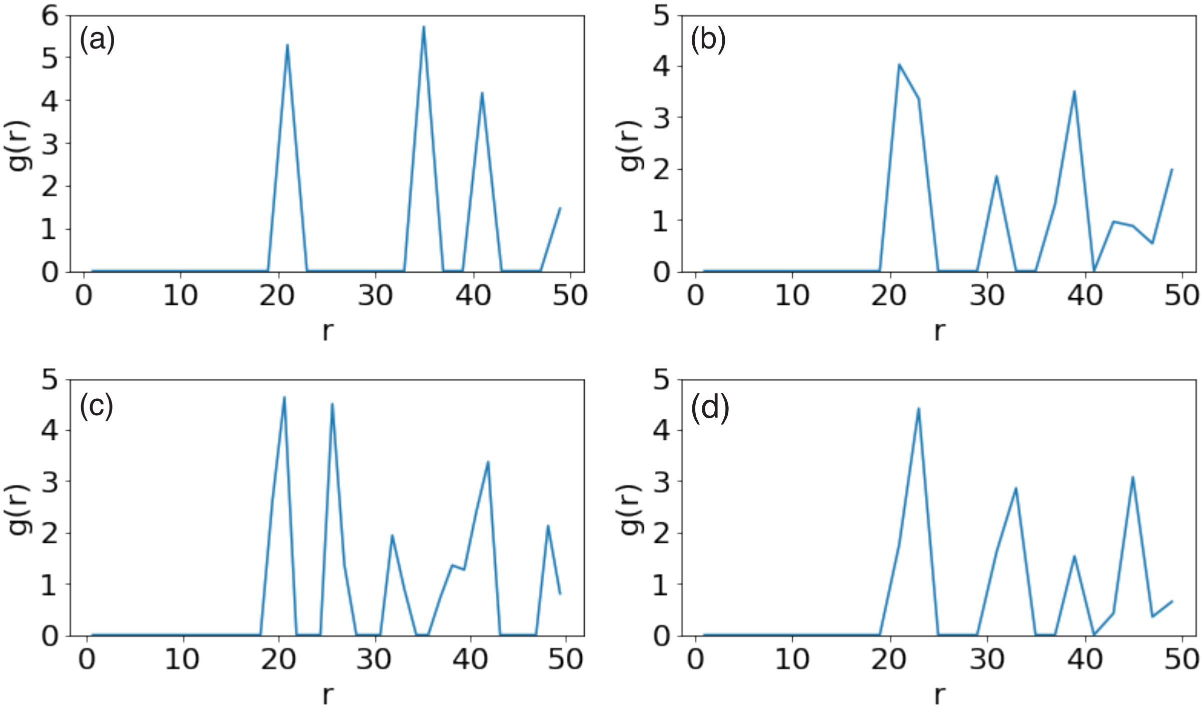

Figure S6: The RDFs used for comparison when colouring the phase diagrams from the window-to-window and window-to-arene simulations. (a) The RDF used in the window-to-window simulation to colour the phase diagram orange (b) the RDF of the red component used to colour the window-to-arene phase diagram which corresponds to the phase where ($\sigma_{ang} = 0.7 - 1.0$) (c) the RDF of the blue component used to colour the window-to-arene phase diagram which corresponds to the phase where $\sigma_{ang} = 0.5$ (d) the RDF of the green component used to colour the window-to-arene phase diagram which corresponds to the phase where $\sigma_{ang} = 0.2 - 0.4$.

6 Arene-to-arene configuration

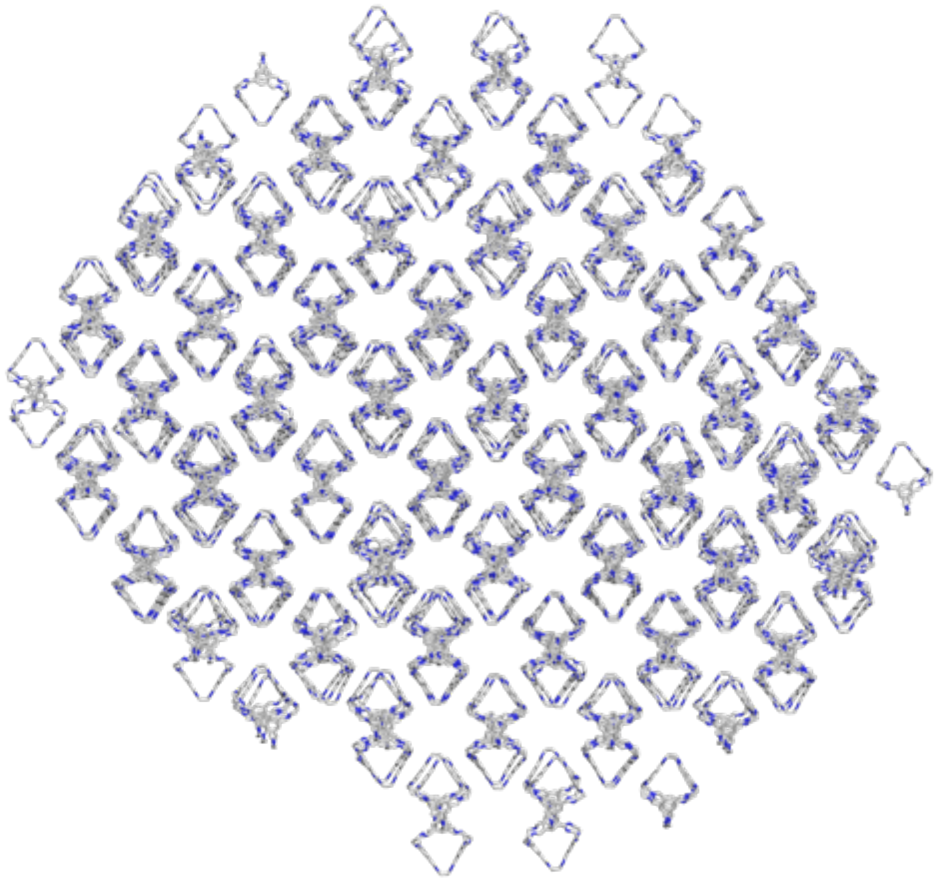

Figure S7: Representative configuration for the arene-to-arene simulations where $\sigma_{ang} = 0.3$.

7 High σ_{ang} leads to disorder

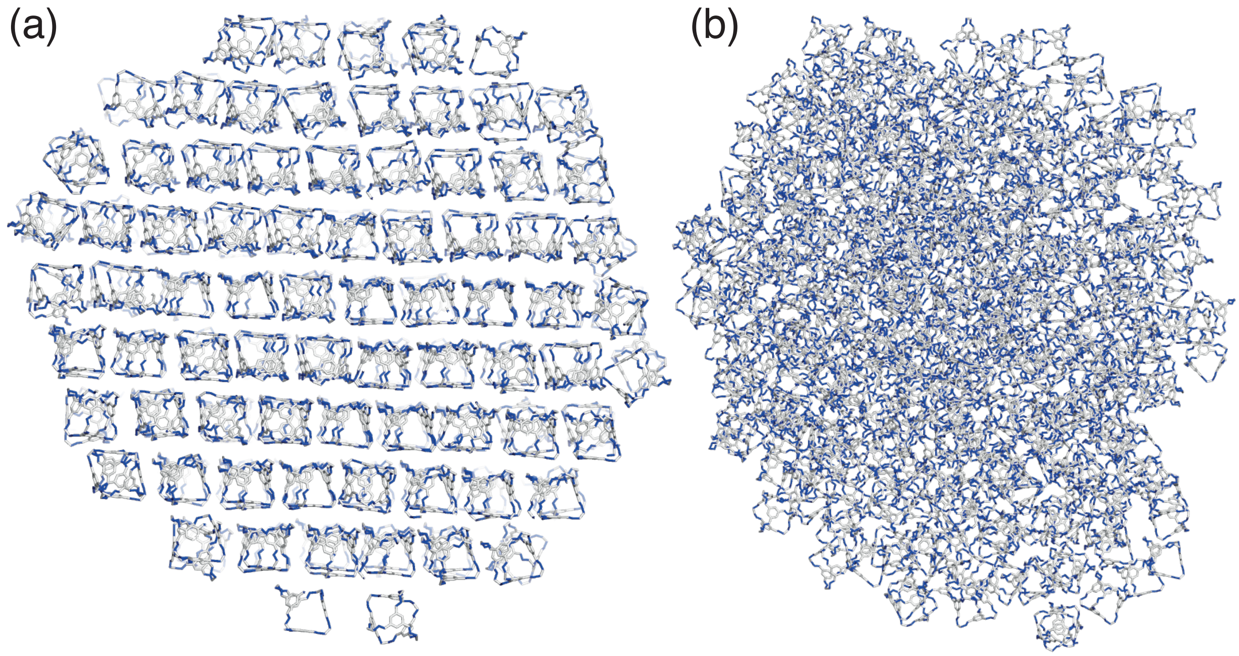

Figure S8: (a) Cross section of a representative configuration for the window-to-arene simulations where $\sigma_{ang} = 0.9$ showing that the orientational behaviour becomes disordered at high σ_{ang} and σ_{tor}. (b) Representative cluster of the amorphous behaviour at high temperature for simulations where $\sigma_{ang} \geq 0.8$. This configuration is taken from the simulation where $\sigma_{ang} = 0.7$. CIF files for both these phases are provided in the ESI.

8 Porous window-to-arene configurations

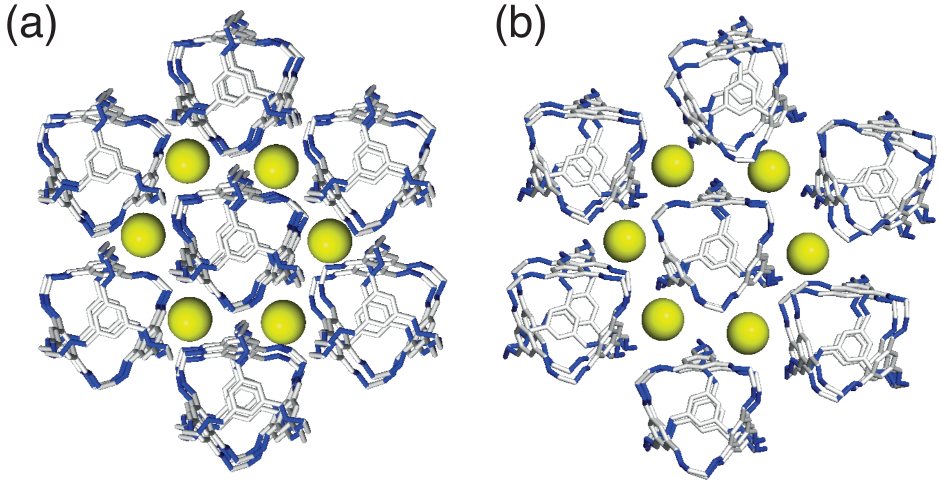

Figure S9: Representative configurations and 1D pore channels (shown in yellow) of the structures from the window-to-arene simulations where (a) $\sigma_{ang} = 0.2 - 0.4$, and (b) $\sigma_{ang} = 0.5$.

9 Predictive capabilities

To determine whether atomistic calculations can be used to determine the likely values of σ_{ang} for known cages, we looked at how the energies of two cages slipping over one another changes as a function of position and distance. For this we focused on the energetics of **CC9** and **CC1** due to them both having solid-state structures which exist within the same simulations; window-to-arene. We first fully optimised both **CC9** and **CC1** in the mixed Gaussian and plane waves code CP2K/QUICKSTEP[S4] with the PBE functional,[S5] GTH-type pseudopotential,[S6] TZVP-MOLOPT basis sets,[S7] using a cutoff of 400 Ry for the plane wave grid and the Grimme-D3 dispersion correction.[S8] With the optimised cages, we than ran single point calculations using the OPLS3 force field[S9] as implemented using Schrödinger[S10] on two cages with the same orientation which were displaced next to eachother along the $\langle 111 \rangle$ axis (Fig. S10(a)). To determine the preferred orientation and displaecment between the two moleclues, we rotated one of the cages around the $\langle 111 \rangle$ octant at different distances from the first cage (Fig. S10(b)). To monitor how the energy of the dimers changed with position, we translated one of the cages around the $\langle 111 \rangle$ octant at different distances from the first cage (Fig. S10textcolorblue(c)). For these calculations we constrained the vertices of the cages so as to ensure we were sampling the energy of the rigid cages textcolorbluerotating/slipping, rather than possible conformational effects. We then measured the interaction energy between the cages in each pair by calculating:

$$E_{int} = E_{pair} - 2E_{cage} \tag{1}$$

For the rotation calculations, we plotted all points on a graph where $E_{int} < 0$ (Fig. S11). These showed that the bulkier side groups considered here would not effect σ_{LJ} and as such our octahedral shapes can be considered a crude representative of all the cages. For the slipping calculations, we then plotted all the energies where $(E_{int} - E_{int}^{min}) \leq 30$ kJmol^{-1} where E_{int}^{min} is the minimum interaction energy between two cages (Fig. S12). We only looked

at energies within 30 $kJmol^{-1}$ as we deemed that energies higher than 30 $kJmol^{-1}$ would be energetically unfavourable. These results showed that **CC1** has more freedom in the slipping motion than **CC9** as there are positions off the central $\langle 111 \rangle$ axis which are within the 30 $kJmol^{-1}$ limit for **CC1**, whereas **CC9** only has on axis low energy configurations. This helps explain why **CC9** is found at a lower value of σ_{ang} than **CC1**.

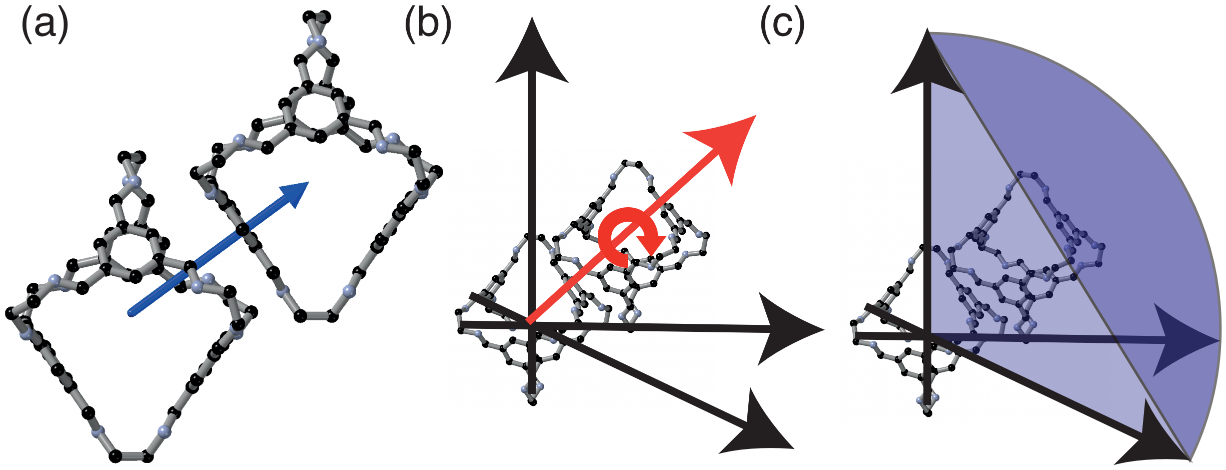

Figure S10: (a) Example of a configuration used in a dimer calculation with **CC1** cages where the cages are displaced along the ⟨111⟩ axis, shown as a blue arrow. (b) Vector (shown in red) that the cage was rotated around and displaced along to examine the effect of rotation and displacement on the interactions between the two cages. (c) Octant (shown in blue) that the cage was displaced around to monitor the effect of cage slipping on the interaction energy.

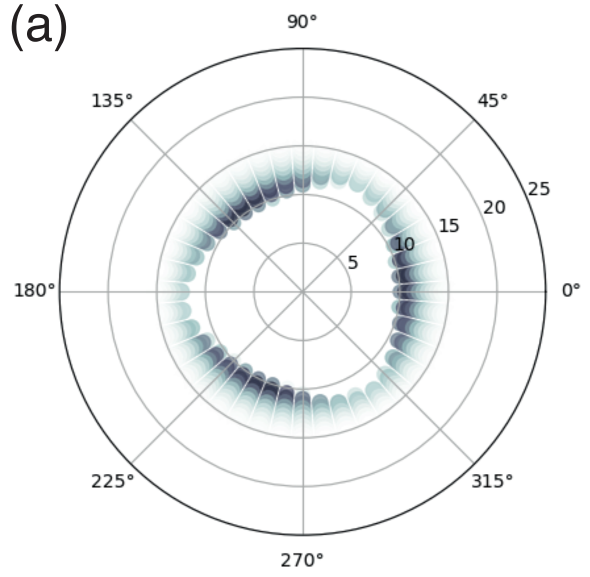

Figure S11: Polar plots showing the energetics of cages rotating around and displacing along the ⟨111⟩ axis for (a) **CC1** and (b) **CC9**. Here r is the distance along the ⟨111⟩ axis and θ is the angle of rotation. The colour of the plot is a measure of the interaction energy where the lower the energy, the darker it is.

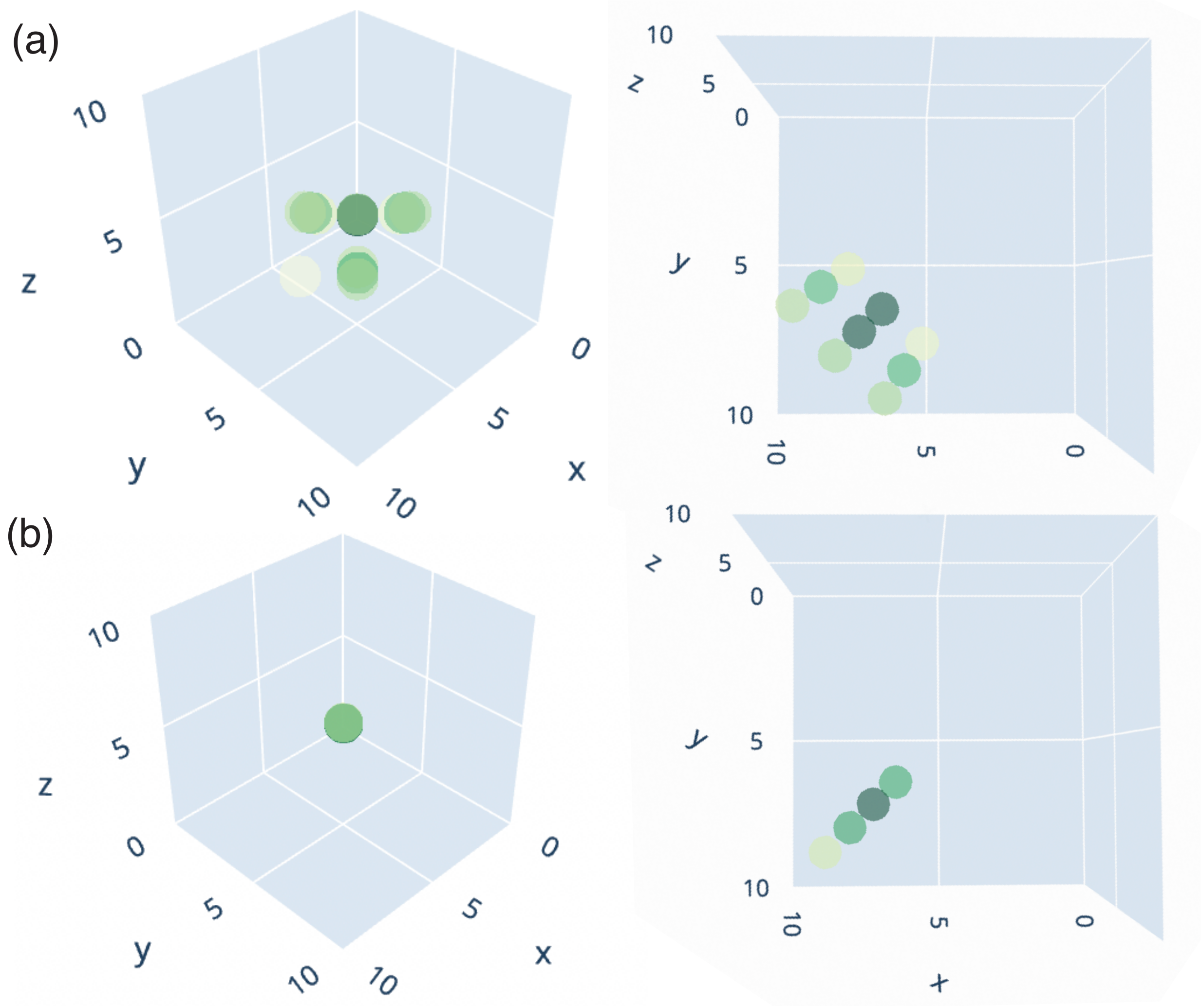

Figure S12: The energetics of cages slipping over eachother for (a) **CC1** and (b) **CC9**. The left hand side is looking down the ⟨111⟩ axis and the right hand side is a cross section containing the lowest energy position looking down the z axis. Points are shown when the energy of the cages slipping at each position is ≤ 30 kJmol^{-1}. The colour of the plot is how close to the minimum the energy is, where the closer to the minimum energy it is, the darker green it is, and the closer to the limit it is, the more yellow it is.

References

(S1) Stokes, H. T.; Hatch, D. M.; Campbell, B. J. FINDSYM, ISOTROPY Software Suite, iso.byu.edu.

(S2) Stokes, H. T.; Hatch, D. M. *FINDSYM*: program for identifying the space-group symmetry of a crystal. *J. Appl. Crystallogr.* **2005**, *38*, 237–238.

(S3) Cui, P.; McMahon, D. P.; Spackman, P. R.; Alston, B. M.; Little, M. A.; Day, G. M.; Cooper, A. I. Mining predicted crystal structure landscapes with high throughput crystallisation: old molecules, new insights. *Chem. Sci.* **2019**, *10*, 9988–9997.

(S4) Kühne, T. D.; Iannuzzi, M.; Del Ben, M.; Rybkin, V. V.; Seewald, P.; Stein, F.; Laino, T.; Khaliullin, R. Z.; Schütt, O.; Schiffmann, F.; Golze, D.; Wilhelm, J.; Chulkov, S.; Bani-Hashemian, M. H.; Weber, V.; Borštnik, U.; Taillefumier, M.; Jakobovits, A. S.; Lazzaro, A.; Pabst, H.; Müller, T.; Schade, R.; Guidon, M.; Andermatt, S.; Holmberg, N.; Schenter, G. K.; Hehn, A.; Bussy, A.; Belleflamme, F.; Tabacchi, G.; Glöß, A.; Lass, M.; Bethune, I.; Mundy, C. J.; Plessl, C.; Watkins, M.; VandeVondele, J.; Krack, M.; Hutter, J. CP2K: An electronic structure and molecular dynamics software package - Quickstep: Efficient and accurate electronic structure calculations. *J. Chem. Phys.* **2020**, *152*, 194103.

(S5) Perdew, J. P.; Burke, K.; Ernzerhof, M. Generalized Gradient Approximation Made Simple. *Phys. Rev. Lett.* **1996**, *77*, 3865–3868.

(S6) Goedecker, S.; Teter, M.; Hutter, J. Separable dual-space Gaussian pseudopotentials. *Phys. Rev. B* **1996**, *54*, 1703–1710.

(S7) VandeVondele, J.; Hutter, J. Gaussian basis sets for accurate calculations on molecular systems in gas and condensed phases. *J. Chem. Phys.* **2007**, *127*, 114105.

(S8) Grimme, S.; Antony, J.; Ehrlich, S.; Krieg, H. A consistent and accurate ab initio parametrization of density functional dispersion correction (DFT-D) for the 94 elements H-Pu. *J. Chem. Phys.* **2010**, *132*, 154104.

(S9) Harder, E.; Damm, W.; Maple, J.; Wu, C.; Reboul, M.; Xiang, J. Y.; Wang, L.; Lupyan, D.; Dahlgren, M. K.; Knight, J. L.; Kaus, J. W.; Cerutti, D. S.; Krilov, G.; Jorgensen, W. L.; Abel, R.; Friesner, R. A. OPLS3: A Force Field Providing Broad Coverage of Drug-like Small Molecules and Proteins. *J. Chem. Theory Comput.* **2016**, *12*, 281–296.

(S10) Schrödinger Release 2022-3: Maestro, Schrödinger, LLC, New York, NY, 2021.
